# Primary and secondary caregiver burden of cognitive impairment associated with schizophrenia: a qualitative study based on caregiver interviews

**DOI:** 10.1038/s41537-025-00675-1

**Published:** 2025-10-17

**Authors:** Sébastien Tulliez, Alessandra Girardi, Matthew Ridley, Katja Rudell, Christoph U. Correll, Abraham Goldring, Claudia Hastedt, Richard S. E. Keefe, Corey Reuteman-Fowler

**Affiliations:** 1 Boehringer Ingelheim International GmbH, Ingelheim am Rhein, Germany; 2 Parexel International, Milan, Italy; 3 Parexel International, Stockholm, Sweden; 4 Parexel International, London, UK; 5 Zucker Hillside Hospital, Department of Psychiatry, Northwell Health, Glen Oaks, NY USA; 6 Donald and Barbara Zucker School of Medicine at Hofstra/Northwell, Department of Psychiatry and Molecular Medicine, Hempstead, NY USA; 7 The Feinstein Institute for Medical Research, Center for Psychiatric Neuroscience, Northwell Health, New Hyde Park, NY USA; 8 Charité - Universitätsmedizin Berlin, Department of Child and Adolescent Psychiatry, Berlin, Germany; 9 German Center for Mental Health (DZPG), Partner Site Berlin, Berlin, Germany; 10 German Center for Child and Adolescent Health (DZKJ), Partner Site Berlin, Berlin, Germany; 11 Nathan S Kline Institute, New York, NY USA; 12 Episteme Inc, New York, NY USA; 13 Duke University Medical Center, Durham, NC USA; 14 Boehringer Ingelheim Pharmaceuticals Inc., Ridgefield, CT USA

**Keywords:** Schizophrenia, Psychosis

## Abstract

Cognitive impairment associated with schizophrenia (CIAS) places a significant burden on patients and caregivers. This study explored the burden experienced by primary (informal) and secondary (formal) caregivers. A secondary qualitative analysis was performed on interviews from a Schizophrenia Cognition Rating Scale (SCoRS) confirmation study. Thematic analysis was utilised to investigate the characteristics of caregiver burden. Quotes were analysed across 3 levels (Level 1: caregiver experience of patient cognitive symptoms; Level 2: caregiver experience of patient burden; Level 3: overarching caregiver burden) to establish themes. Subsequently, themes were mapped to SCoRS domains. Twenty primary caregivers and 20 secondary caregivers were enrolled. Caregivers described patient difficulties with memory, learning, social communication, and everyday tasks. Caregivers assisted patients by engaging in sustained teaching for new activities, encouraging social communication and repeating reminders to accomplish tasks. Caregiver burden was substantial among both groups. Thematic analysis identified 2 main themes: caregiving difficulty and caregiving necessity. These themes reflected the extensive roles and responsibilities assumed by caregivers due to the impacts of CIAS symptoms. Illustrative quotes were reported for 60% of primary caregivers and 55% of secondary caregivers. Six of the 8 SCoRS domains (memory, learning, attention, problem-solving, language, social cognition)were mapped to themes identified at each level. A high level of objective caregiving burden was found in relation to CIAS symptoms among primary and secondary caregivers. The SCoRS domains of memory, learning, attention, problem-solving, language and social cognition are key areas to treat in patients with schizophrenia to reduce caregiver burden.

## Introduction

Schizophrenia is a complex, chronic psychiatric disorder that presents many challenges; impairments in daily functioning and quality of life can be difficult for patients to manage, and the impact of these challenges often extends to caregivers^1–3^. In addition, the role of society in supporting patients with schizophrenia entails the provision of healthcare services, such as long-term psychosocial care, inpatient and outpatient hospital visits/stays, as well as economic support, resulting in high direct and indirect costs^1–3^. Although prevalence estimates of schizophrenia show variation, a systematic literature review that included data from 46 countries reported a median lifetime morbid risk of 0.72%, which means that about 7 individuals per 1000 will be affected^4^. The disorder typically manifests in late adolescence or early adulthood, with onset occurring earlier in men than in women^3,5^.

Schizophrenia is characterised by a combination of positive, negative and cognitive symptoms^6^. Positive symptoms, such as delusions and hallucinations, are typically the initial reasons for clinical presentation^6^. Negative symptoms, including amotivation and social withdrawal, and cognitive symptoms such as deficits in working memory, executive function and processing speed, contribute to the disorder’s long-term burden^6^. Cognitive symptoms are often present at the first signs of schizophrenia, affecting approximately 80% of individuals with the condition^7,8^. Cognitive impairment associated with schizophrenia (CIAS) affects daily life and is a major determinant of functional outcomes^7^, as well as affecting healthcare costs^9^.

The EU5 National Health and Wellness Survey found that schizophrenia caregivers reported greater activity impairment, resource utilisation, costs and worse health-related quality of life than non-caregivers and caregivers of other conditions^10,11^. The substantial costs associated with schizophrenia, including caregiver healthcare needs, productivity loss, and reliance on caregivers, have led Health Technology Assessments, payers and prescribers worldwide to recognise the value of evidence demonstrating alleviation of caregiver burden^12^. Assessing caregiver burden in clinical trials and practice can enhance awareness and management, offering opportunities for increased support that may reduce the direct and indirect costs associated with caregivers’ healthcare needs, loss of productivity and reliance on formal caregivers^12^. Improving family and social support systems may reduce the burden on schizophrenia caregivers and healthcare systems, ultimately decreasing the societal cost of schizophrenia globally^10^.

Informal caregivers (i.e., primary caregivers) for patients are unpaid family members, friends or neighbours involved in daily care, whereas formal caregivers (i.e., secondary caregivers) are paid professionals, for example, psychiatrists, nurses or social workers, and are likely to be unrelated to the patient. Caregivers experience substantial economic impacts, including greater absenteeism, presenteeism, overall work impairment, and indirect costs associated with their caregiving roles compared with non-caregivers^10^. The financial burden for caregivers can be significant, with annual average costs ranging from $1586 in China to $30,591 in the US^13^. While research shows that primary caregivers are affected emotionally, financially and physically, experiencing restricted daily routines and by other burdens such as stigma, blame and dissatisfaction with family dynamics^14^, secondary caregivers have so far received minimal attention in research. Nevertheless, secondary caregivers are a relevant group that is involved in the care of people with schizophrenia, and a previous study that included secondary caregivers and that focused on economic impact has shown that there is at least an economic cost to secondary caregivers of people with schizophrenia^13^.

The Schizophrenia Cognition Rating Scale (SCoRS) is an interview-based measure designed to assess cognitive impairment and impact on day-to-day functioning in the real world in patients with schizophrenia, with questions relating to specific cognitive domains, such as memory, learning, attention, working memory, problem solving, speed of processing, language and social cognition^15,16^. Assessment with the SCoRS requires input from the patient, the caregiver and the clinician. Prior studies have shown SCoRS to have strong psychometric properties, with excellent test-retest reliability and that it is highly related to cognitive performance (per the MATRICS Consensus Cognitive Battery), in addition to being sensitive to treatment^16^.

One recent study, the SCoRS concept confirmation, further established the relevance of the content and usability of the SCoRS from the perspective of both primary and secondary caregivers looking after patients with schizophrenia. The study reported here is a secondary analysis of the primary SCoRS concept confirmation study and had the following objectives: first, to identify primary and secondary caregiver burden experience in relation to CIAS when caring for patients with schizophrenia, based on data collected during the SCoRS content confirmation study^17^; second, to describe core themes related to caregiver burden of CIAS; and third, to identify SCoRS domains that are related to these themes to determine which domains can be targeted with new treatments to alleviate caregiver burden of CIAS.

## Methods

### Study design

This study employed a secondary, qualitative, inductive thematic analysis using qualitative interviews from the SCoRS concept confirmation study^17^. The primary study was a qualitative, cross-sectional, non-interventional study conducted in the US, in which caregivers who cared for patients with schizophrenia participated in one-on-one, 90-minute, semi-structured interviews conducted by trained researchers^17^.

Approval for primary data collection was granted by the Salus Institutional Review Board. All participants provided written informed consent prior to participating in interviews and provided consent for secondary analyses.

### Participants

In the primary (SCoRS concept confirmation) study, caregivers were recruited through US patient/caregivers and professional association groups, or via clinical trial sites if the caregiver did not care for a patient taking part in an ongoing clinical trial. A total of 50 primary and secondary caregivers of patients with schizophrenia were identified between October 2021 and March 2023. Caregivers were included in the study if they were ≥18 years old, knew the patient well, regularly interacted with them (≥1 hour/week with ≥1 in-person interaction/week) could read, write and speak English, and exhibited reliability and physiologic capability (e.g., sufficient hearing and vision) to comply with protocol procedures (in the investigator’s opinion). In addition, primary carers were required to be educated to at least 8th grade and secondary carers needed to have formal training as a health or social care professional. The final sample size was 40 caregivers (20 primary caregivers; 20 secondary caregivers). Most primary caregivers were female (85%), with a mean age of 51 years (range 28–82), while secondary caregivers also showed a predominance of female participants (60%), with a mean age of 41 years (range 22–60). Further details on caregiver demographics and characteristics are reported in the SCoRS concept confirmation study^17^.

Primary caregivers were defined as individuals providing informal (unpaid), live-in care to the patient, offering direct or indirect support such as organising tasks, making phone calls, managing bills or paperwork, shopping, cooking, cleaning and doing laundry. Secondary caregivers were defined as individuals with formal training who worked with patients with schizophrenia in outpatient and/or home settings, such as case managers, group home staff members, psychiatric social workers, social workers and psychiatric mental health nurses.

### Measures

The 20-item SCoRS is an interview-based assessment that evaluates cognitive impairment and the impact of cognitive impairment on the daily functioning of patients with schizophrenia. The SCoRS measure includes 8 cognitive domains: memory, learning, attention, working memory, problem-solving, processing/motor speed, language and social cognition. Items are rater-assessed (a final rating based on the clinical judgement of the interviewer with input from patients and caregivers) on a 4-point scale; higher scores indicate a greater degree of cognitive impairment^15,17,18^.

### Procedures

Interviews with caregivers were conducted remotely via video call using Microsoft Teams, and the audio was recorded and subsequently transcribed. Transcripts were de-identified prior to being uploaded to ATLASti^TM^ software package for the primary data analysis.

Two discussion guides were used in the interviews; 1 for primary caregivers and 1 for secondary caregivers (Supplementary methods). The interviews were structured in 3 parts: caregiver and patient characteristics, concept elicitation, and cognitive debriefing of the SCoRS. The results of the concept elicitation and cognitive debriefing are reported in the SCoRS concept confirmation study^17^. Interview questions included caregiver demographics and characteristics, caregiver perception of the patient’s CIAS severity (none, mild, moderate or severe) in the previous 2 weeks, caregiver experience of caring for or spending time with the patient with schizophrenia. Interviews investigated the relevance of the SCoRS content to assess the impact of cognitive functions on the patient’s daily life and were not designed to directly ask about the caregiver’s experience of caring for patients with schizophrenia.

### Data analysis

**Fig. 1:**
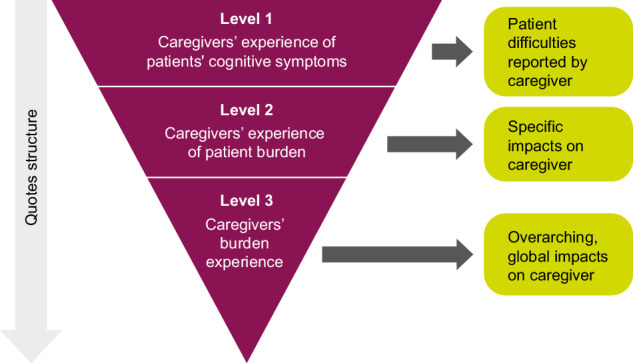
Caregiver analysis data levels.

Inductive thematic analysis was selected as the optimal approach for data analysis^19^, following a search within the International Society for Pharmacoeconomics and Outcomes Research (ISPOR) database to identify qualitative methodologies focusing on participant experience. This method is widely used in psychology and social sciences for reporting themes within data and offers a highly flexible approach to analysing participant experience data^19^. In this secondary analysis, data were extracted from interview feedback provided by the 40 participating caregivers, comprising 20 primary and 20 secondary caregivers, during the SCoRS content confirmation study. Quotes from the interviews were analysed across 3 levels (Level 1: caregiver experience of patient cognitive symptoms; Level 2: caregiver experience of patient burden; Level 3: overarching caregiver burden; Fig. 1). A separate inductive thematic analysis was conducted for each level, with codes assigned and themes established at each step. The process was iterative, moving between the levels to establish the 3 sets of themes. The analysis was conducted by an individual researcher, whose work was then reviewed by a second, senior individual researcher. Any disagreements were resolved with consensus between the data analyst and the senior researcher. Themes from each level were mapped to SCoRS domains (memory, learning, attention, working memory, problem-solving, processing/motor speed, language and social cognition) to indicate which cognitive domains are relevant to the burden experiences of caregivers.

## Results

### Caregiver rating of patient CIAS burden

In the 2 weeks prior to the interviews, a similar proportion of primary caregivers rated patient cognitive impairment as mild, moderate or severe, with only 10% reporting patients as having no impairment. In contrast, secondary caregivers mainly reported patient cognitive impairment as moderate and none reported it as severe. Care settings varied, with 25% of patients with primary caregivers in outpatient settings, 25% of patients in home care and 50% in community settings. Among patients with secondary caregivers, 55% were in outpatient settings, 30% in inpatient settings, 25% in home care, and 5% in community settings (some patients had care in more than one setting). Hospitalisation for schizophrenia in the past year was reported for 30% of patients with primary caregivers and 40% with secondary caregivers. The mean duration of hospital stays during the past year was longer for those with secondary caregivers, at 18.3 weeks compared with 1.7 weeks for primary caregivers. Further details are reported in the SCoRS concept confirmation study^17^.

### Caregiver burden findings

#### Level 1: Caregiver experience of patient cognitive symptoms

Four themes concerning the caregiver experience of patient symptoms were identified. These themes were: problems with memory, notably issues remembering names, appointments and frequent misplacing of items; difficulty learning new things, characterised by slow learning and the necessity for repeated, one-on-one instruction; difficulties with social communication, including a lack of response to social cues and difficulties engaging in group conversations; and finding it hard to fulfil everyday living activities, such as finance management, cooking and personal care routines (Table 1).

**Table 1:** Level 1 theme descriptions and illustrative quotes from primary and secondary caregivers.

Theme	Description	Illustrative quotes	
		Primary caregiver	Secondary caregiver
**1. Problems with memory**	Problems remembering names (own, others, the caregiver), appointments, places, dates and times; losing/misplacing things	“Basically, he has a lot of problems, let’s say, with memory. **He comes to a point when he can’t even remember the names of the family members sometimes**, and there are a lot of hallucinations.” (P14) “There was a time my aunt visited and **she could not remember her name**.” (P15)	“He and I have been working together for the past two years and **he often times forgets my name, particularly my last name**. Phonetically, he can say my name, but he wouldn’t be able to spell it. If this makes any sense, he understands the rhythm of my name.” (S3) “Sure, so with the clinic, it’s me, I’m [Name], there’s [Name], and there’s [Name] and [Name]**. He remembers none of those names. It’s four of us. None of us look alike.”** (S14)
**2. Finding learning new things difficult**	Takes a long time to learn; 1:1 teaching required; poor concentration/focus	“You said learning is part of cognitive. And then you will find also, if it’s a game you are teaching her, you’ll have to repeat so many times such that… My kid who is 6 years old can even capture the concept more than she does. **So, it will take a long time for her to learn how it is being played and to play on her own. So, I can say she learns very slowly**.” (P12)	“Learning to do activities here, it may be something new for him, or transition, schedule changes. **In order for him to be comfortable, or to get him to follow through and be cooperative, it really would need to be someone that directly works with him, that’s helping him to learn this or to make that change. Or if not, he’ll refuse and he won’t do it. ”** (S19)
**3. Difficulties with social communication**	Doesn’t pick up on social cues; prefers to be alone; difficulty talking in groups; questions and commands need to be broken down/rephrased/repeated	**“Currently, she pretty much spends all her time alone**. It’s what she prefers. We do find when we try to interact with her, **she has a canned response to us, so what she has developed is always pretty much the same, therefore she doesn’t really have to listen to what we say and process it**, she just blurts out, so even if you ask a question, you’re still going to hear the same canned response. **We try to slow her down and say, “No, this is what we are asking you, we need an answer for this.” I guess maybe you need to ask me again what you want.”** (P16)	“(…) just if there’s more – **even with just one person talking to him, you often have to repeat yourself, rephrase questions, break them down into easier to remember terms. And if he’s in a room with more than one person, if all three or four people are talking, he often gives you like a blank stare, or cannot kind of grasp what the conversation’s about. If a question is asked of him, he’ll just kind of look at you and he’ll need to be walked through the question.”** (S12)
**4. Finds it hard to fulfil everyday living tasks/activity**	Finance budgeting; cooking; self-care; difficulties with daily routine – maintenance and understanding	“Having to maybe change a schedule where he has to go somewhere at a different time. (…)**. Any type of change involving having to let’s say even get dressed in the morning. If you change the time that he normally would get up to a different time, and just getting on that schedule might be a little bit difficult for him to do**.” (P18) “I don’t know if this is important or not. **He has a hard time cooking for himself**. I just think it’s hard for him with all the medications he’s on and working nights, but I don’t know if that is anything that would be helpful. But I know to teach him to cook, it’s kind of hard to do.” (P9)	“First thing that stands out is **just self-care, remembering to take baths, brush his teeth, change clothes**. He can start a task with assistance, but it’s hard finishing tasks. But we do transport our clients, and so it’s better now.” (S19) “Sometimes **they don’t have ability to balance out their daily routine**. Like I said, they don’t keep up their appointments because they forgot they had their appointment or they didn’t realize that they needed to come on a certain day.” (S2)

P + number denotes the primary informant identification number. S + number denotes the secondary informant identification number. Main concepts from the quotations are marked in bold.

#### Level 2: Caregiver experience of patient burden

Four themes were identified concerning the caregiver experience of patient burden. The themes were “Support required for memory recall”, where caregivers provide prompts, reminders and assist with remembering names and places; “New activities/technology use requires sustained teaching assistance”, indicating the need for consistent one-on-one instruction; “Multiple elements of social communication assistance are required (e.g., support for the patient to listen, follow, respond and express)”, highlighting challenges in helping patients engage in and follow conversations, especially in group settings; and “Everyday living tasks require repetitive verbal support”, where caregivers assist with tasks related to finance management, cooking, self-care and maintaining a daily routine (Table 2).

**Table 2:** Level 2 theme descriptions and illustrative quotes from primary and secondary caregivers.

Theme	Description	Illustrative quotes	
		Primary caregiver	Secondary caregiver
**1. Support required for memory recall**	Assistance includes prompts, reminders, phone calls, post-it notes; and with names and places	“So he also **struggles to remember to keep his appointments with his healthcare providers**. He does seem to not forget when I’m coming, because I take him shopping every week, and that he doesn’t forget about. But when he has other appointments, he often forgets about those.” (P8)	“Oh, yeah. Well, I mean, just directly, we would have an appointment scheduled for, say, Thursday at 9:00 a.m. and 9:05, 9:10, they’re not here. 9:30, okay, we call. **Even though they had already received a call the day before, that they had the appointment at 9:00, but that they had forgotten about the appointment**. So, usually, the subject’s been okay coming in afterwards, but they had to get a second call from us to remind them that they had already missed their appointment, and this has happened for numerous appointments.” (S12)
**2. New activities/technology use requires sustained teaching assistance**	Consistent 1:1 teaching required by the caregiver	“You said learning is part of cognitive. And then you will find also, if it’s a game you are teaching her, you’ll have to repeat so many times such that… My kid who is 6 years old can even capture the concept more than she does. **So, it will take a long time for her to learn how it is being played and to play on her own. So, I can say she learns very slowly**.” (P12)	“There is an app that’s related to the investigatal^1^ product that we give to the patients, and there’s a device that we hand out in the beginning of the trial. And the **subject has many issues using the device, working a device, and actually using it on a daily basis as he is required to**. So, there’s been many instances where if **I had to re-educate the subject on how to use the device**, and there 9, 10 months that he was in the trial, it progressively got worse. And then at one point, he was pretty non-compliant with not using the actual device.” (S10)
**3. Multiple elements of social communication assistance are required (e.g., support for the patient to listen, follow, respond and express)**	Help with following, contributing to, and ultimately, being able to express themselves in conversations with others – particularly in a group	“Well, for her to be **more independent and more organized for the future she still – she’s vague lots of times about interactions** with like the therapist or the psychiatrist or whoever she’s working with in a vocational program about the details of what she needs to do or what was said. **I get maybe half or three-quarters of the story and then I have to follow up to make sure that things are taken care of or that they happen**. That kind of thing. She doesn’t really write them down like in a calendar or anything like that too much. So those kinds of organizational things. However, if it was where an apartment was and the address and setting up an apartment, yeah I think she can do that.” (P5) “I have noticed because, like I said before, maybe she’s hungry. She needs something to eat. **But whatever she’ll come and tell you, it’s absolutely something totally different**. So you being the person, being the caregiver, you have to figure these things out for yourself. You just have to figure it out on your own. If she’s asking for something, it’s like a baby. Maybe she’s hungry. Maybe she wants to go to the toilet, or maybe she wants to go out. So, you have to battle all between those things, and figure out what she wants. So, you try and give her that. If she doesn’t want that, if that’s not what she wanted, you switch to something else until you finally get what she really wants**. But having stayed with her for quite some time, you have a way of understanding them**.” (P13)	“(…) **I don’t think he does have a good connection to how the other people around him feels when he’s expressing himself in certain areas**, because he just – he will be just communicating, and talking, and saying things without allowing the other person to kind of engage with him, in a way, unless you interrupt and say, “Hey, you know,” kind of interrupt him a little bit.” (S7) “**It’s just bothersome because they need to be able to express themselves so people will know what they’re talking about, so that they can get the help that they need if they’re seeking help**. If you can get them in a group that’s like a one on one, you’re able to assist them. And that’s not going to always be the case. That’s the only thing bothersome about that, **is we’re trying to get them engaged within that group setting**, so they can be more comfortable, and pay attention, and try to focus on what’s being said in that group setting.” (S18)
**4. Everyday living tasks require repetitive verbal support**	Difficulties include those related to finance budgeting, cooking, self-care; Issues with daily routine – maintenance and understanding	“I can routinely ask him about something that I may have asked him to do. For instance, I can say, did you feed the dog? And then he’ll go**, “mmm…yes.” It’s that much delayed, or maybe even longer sometimes, because I may have to repeat myself**.” (P18)	“They get very frustrated. They **get a little agitated. If they don’t know how to do certain things, they feel embarrassed**. They try to make light of it, making excuses or try to avoid certain things. I think that’s **maybe a reason why they don’t keep up with their appointments sometime because they forgot about the appointment, even though they were reminded about the appointment the day before coming**. When I go and get them, they’re not ready. And even when I call and say, “I’m on my way”, they’re still not ready because they forgot that I was coming.” (S2)

P + number denotes the primary informant identification number. S + number denotes the secondary informant identification number. Main concepts from the quotations are marked in bold.

#### Level 3: Overarching caregiver burden

**Fig. 2:**
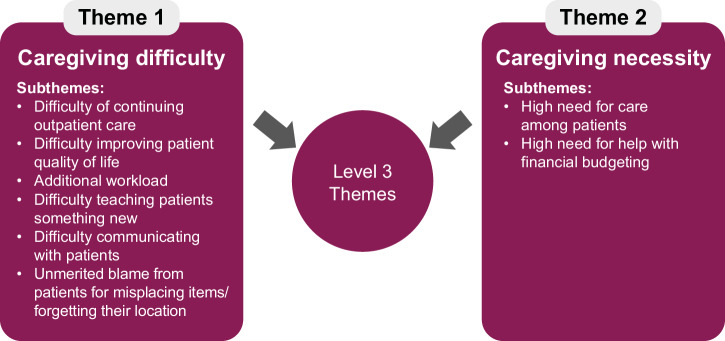
Thematic map of caregivers experience of burden.

The final overarching analysis identified 2 main themes, ‘caregiving difficulty’ and ‘caregiving necessity’, both shown in Fig. 2. Caregiving difficulty and caregiving necessity (distinct but related topics) were both depicted as high. Consequently, a substantial level of objective caregiver burden was reported across the data set for both primary and secondary caregivers. ‘Caregiving difficulty’ includes subthemes such as the difficulty of continuing outpatient care, the effort to improve patient quality of life, additional workload, teaching new skills to patients, and the frequent need to communicate repeatedly. ‘Caregiving necessity’ highlights the high need for caregiver help in daily activities and financial management, emphasizing the patient’s dependence on caregiver support for basic functioning and safety. Illustrative quotes that provide real-life examples of these themes are shown in Table 3 for ‘Caregiving difficulty’ and in Table 4 for ‘Caregiving necessity’.

**Table 3:** Level 3 Theme 1 Caregiving difficulty illustrative quotes.

Primary caregiver experiences	Secondary caregiver experiences
**Improving patient’s quality of life**: “**Well, for me, I’m trying to get him a quality of life and it’s very difficult to give him any kind of an enjoyable experience when he can’t have more than like a 20-minute experience**. So, if we go to the museum, in 20 minutes he wants to leave. If we go to the zoo, in 20 minutes he wants to leave. I spend my day driving him around to have these little 20-minute experiences. It’s really hard to keep his life enriched and not have him just sit and when he’s not doing anything, he’s really bored. So, it’s not like he can enjoy not doing anything either. I don’t know if that makes any sense. **It’s hard to keep his quality of life up with the lack of attention that he has**.” (P2)	**Continuing outpatient care**: “It’s just the fact that **it’s hard to continue outpatient care sometimes because they miss their appointments**. Sometimes, they miss their medications or don’t follow through the regime of the doctors for their medication intake. Yeah, couldn’t in the sense of remembering their appointments, or remembering to take their meds, or wanting, by the way, that’s another thing, but I don’t think that’s part of the study. This particular client doesn’t necessarily want to take medication, so that is a struggle…” (S7)
**Extra responsibilities**: “He forgets, **if you tell him to do something he will forget it**.” (P2) “Well, the thing that’s the **most bothersome to both of us is repeating the same thing over and over** and over and over and over, over and over and over.” (P4)	**Additional workload**: “**Well, an example would be like when he went into the hospital, and this happens to a lot of my clients while hospitalized, they lose the doc, they lose their cards, okay? So, he lost his SNAP card. So, they discharge him without the SNAP card. Now, he can’t access his food stamps. Now, he can’t get food. So, without that SNAP card, I have to reach out to the ICM, to the residence, get him food pantries until we can that activated**, and to me, it’s that kind of benefit, when the housing, food, when they start to not be able to access that, that’s very troublesome.” (S1) “Oh, yeah. Well, I mean, just directly, we would have an appointment scheduled for, say, Thursday at 9:00 a.m. and 9:05, 9:10, they’re not here. 9:30, okay, we call. **Even though they had already received a call the day before, that they had the appointment at 9:00, but that they had forgotten about the appointment. So, usually, the subject’s been okay coming in afterwards, but they had to get a second call from us to remind them that they had already missed their appointment, and this has happened for numerous appointments**.” (S12) **Extra work can be in vain**: “Or the one time they did call they **did not get through and then lost the post-it again**. And this has happened will happen.” (S6)
**Blame for misplacing or forgetting locations of belongings:** “He constantly loses things because he doesn’t remember where he put it. He’s constantly losing things to the point where he blames me for having moved some things that I never touched because he doesn’t even remember that he put it where it is.” (P1)	
**Teaching something new**: “You said learning is part of cognitive. **And then you will find also, if it’s a game you are teaching her, you’ll have to repeat so many times such that… My kid who is 6 years old can even capture the concept more than she does. So, it will take a long time for her to learn how it is being played and to play on her own. So, I can say she learns very slowly**.” (P12)	**Teaching something new**: “What’s most difficult is that as **he’s not able to learn and we’re not able to teach him in a way that he can absorb he misses out on so many things**. He’s not able to manage a phone, a tablet, a non-technological smartphone. He misses out because he can’t do things that other patients can do, so he’s not chosen for certain activities or groups. He misses out.” (S6)
	**Communicating**: “**It has to be okay if I’m trying to engage in an important conversation that he follows me**. That’s important. But if it’s a conversation about – if he’s having a different conversation about, let’s say, the sky is red I will say, “Oh, it is? Okay, that’s interesting. I wonder what colour it’ll be tomorrow.” I won’t challenge him on that, but **it can be frustrating to be in two different conversations at once when the person is either ill or unable to focus on what we’re talking about**.” (S8)

P + number denotes the primary informant identification number. S + number denotes the secondary informant identification number. Main concepts from the quotations are marked in bold. SNAP provides nutritional support to people with low-income by supplementing their grocery budget^29^. ICM provides support, guidance, and coordination of care to meet an individual’s health needs^30^.

ICM, integrated care/case manager; SNAP, Supplemental Nutrition Assistance Programme.

**Table 4:** Level 3 Theme 2 Caregiving necessity illustrative quotes.

Primary caregiver experiences	Secondary caregiver experiences
**Struggling to complete tasks**: “Yeah. So for instance, **if I wasn’t living with him, and if I didn’t set up reminders, and if I didn’t call to make his appointments to go in and get his shot, I don’t think that would get done**. And then he – and if that doesn’t get done enough, he doesn’t – he misses his shots in, let’s say consecutive months, he can go back into psychosis and have a very bad experience. So it’s so important to remember when your shot is, remember when your doctor’s appointment is because that affects your health.” (P6)	**Struggling to complete tasks:** “**His inability to respond, again, in times of danger**. Like if there’s an issue with another patient, if there’s an issue with himself where a patient is harassing him or trying to fight him he won’t be able to really tell us unless we really sit with him for like a good 20, 30 minutes, which is sometimes very difficult to do. **Just to tell him, hey, I know something’s going on, I’m going to sit here and wait with you until you’re able to tell me**. I’m very grateful that I have the ability to do that, but sometimes I don’t. **I’m always worried that if I don’t try to give him the enough amount of time that he might not be able to say or communicate what’s really going on**.” (S6)
**Financial budgeting**: “Yes, he can keep track of money, he can count change. **He’s not good with money, he spends money a lot. He spends it very foolishly**, but I think he can definitely count it and make change.” (P6) “**Him giving his money away**.” (P18)	**Financial budgeting**: “So, like most of us know to budget our money. We know you have to pay this money for this bill and that bill. **This individual would just spend all their money up**. Not understand that you might need money for tomorrow or not all the money is for you. You have to have money for your rent, you need money for food, you need money to buy clothes and other things. **They don’t understand that they need to save some money so they will spend all their money up in one shot**.” (S2)

P + number denotes the primary informant identification number. S + number denotes the secondary informant identification number. Main concepts from the quotations are marked in bold.

Across all 3 thematic analysis levels, illustrative quotes were identified for 12/20 (60%) primary caregivers and 11/20 (55%) secondary caregivers from the interviews in the SCoRS concept confirmation study.

### Mapping of thematic findings onto SCoRS domains

**Fig. 3:**
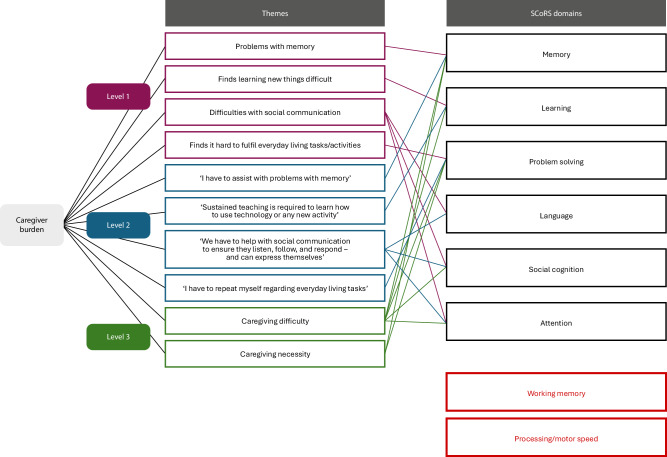
Mapping of caregiver’s burden thematic findings onto SCoRS domains. SCoRS Schizophrenia Cognition Rating Scale.

Six out of 8 SCoRS domains—memory, learning, attention, problem-solving, language and social cognition—were identified as relevant to the caregiver burden reported by both primary and secondary caregivers (Fig. 3). Specific caregiving challenges mapped to these domains include difficulties with daily living tasks, communication and teaching new skills. The working memory and processing/motor speed SCoRS domains were not identified to be relevant to caregiver burden.

## Discussion

This study identified a substantial level of burden among this sample of primary and secondary caregivers for patients with CIAS. Caregivers reported the need to assume extra role responsibilities in relation to the patients’ CIAS symptoms, which was central to their extent of burden. The final thematic analysis identified caregiving difficulty (Theme 1) and caregiving necessity (Theme 2) as the 2 main themes. Illustrative quotes were provided by an equal proportion of primary and secondary caregivers. Memory, learning, attention, problem-solving, language and social cognition were identified as SCoRS domains relevant to caregiver burden.

Primary caregivers reported a more evenly distributed severity (ranging from none to severe) of CIAS symptoms in patients 2 weeks prior to the interview compared with secondary caregivers, who reported only mild or moderate CIAS symptoms. However, it should be noted that the nature of secondary caregiver provision is likely the result of recent and/or ongoing hospitalisations; secondary caregivers reported higher hospitalisation frequency and duration over the past year and 30% were reporting on current inpatients. Therefore, secondary caregivers may have a more clinical approach and definition of severity compared with primary caregivers who are usually exposed to a broader range of behaviours and challenges. Future research should refine the matching of the caregiver groups in relation to the recall period and time of patient CIAS symptom presentation.

The main themes identified were caregiver difficulty (Theme 1) and caregiver necessity (Theme 2). These findings are in accordance with an existing study that identified daily hassles (i.e., assumption of extra responsibilities/caregiver necessity), emotional burden, and financial troubles among the main areas of caregiver burden^14^. Other interview-based qualitative studies also found heavy involvement of caregivers (i.e., caregiver necessity) and challenges in caregiving (i.e., caregiver difficulty) as key themes^20,21^. A correlational study reported that caregivers had a substantial burden, with 38.2% of primary caregivers experiencing severe burden^22^. The caregiver’s age, gender, education level, job loss due to caregiving responsibilities, income, relationship with the patient, disease duration and frequency of caregiving were identified as statistically significant predictive factors for the caregiving burden in schizophrenia^22^. Unlike previous research^14^, physical aspects were not reported in the present study. The themes identified at each level were mapped to 6 of the 8 SCoRS domains, including memory, learning, attention, problem solving, language and social cognition. Therefore, these domains may serve as potential outcome areas in clinical trials to assess the impact of schizophrenia treatments on caregivers.

The burden experienced by caregivers in relation to cognitive impairment among people with schizophrenia demonstrated in this analysis, highlights an unmet need for treatment of CIAS. Current recommendations suggest patients with schizophrenia should be administered second-generation antipsychotics to manage their cognitive symptoms, as there is a favourable cognitive profile as compared with first-generation antipsychotics^23^. Furthermore, psychosocial interventions such as cognitive remediation and physical exercise have been specifically demonstrated to provide some benefit to cognition in people with CIAS^23–28^. Though these approaches may ameliorate cognitive symptoms to some extent, there are no US Food and Drug Administration (FDA)-approved pharmacological treatments specifically targeted for cognitive symptoms. This unmet need will be critical to fill as CIAS is associated with poor functional outcomes, poorer quality of life, and increased healthcare resource utilisation than those without cognitive impairment, in addition to the impacts on and costs incurred by caregivers^1–3,7,9,10,13^.

The study presented several limitations that should be considered when interpreting the findings. First, the secondary nature of the study is the main limitation: the study used pre-existing interview data from the SCoRS concept confirmation study with a different objective. Therefore, the conclusions should be interpreted in respect to this limitation as specific questions about caregiver burden were not asked in the interviews. Nevertheless, the study was rigorous due to the application of an inductive thematic analysis that analysed quotes iteratively across 3 levels, thereby the results provide preliminary information that can be used to design a prospective approach in future studies. Second, the impact of issues related to working memory and processing/motor speed on caregiver burden may not have been adequately captured owing to the design of the study interviews (direct questions were not asked). This approach may lead to the incorrect conclusion that these domains are not relevant. Therefore, further studies are needed to explore these aspects more thoroughly. Third, no evidence emerged on physical aspects of caregiver burden (e.g., somatic issues, less energy, tiredness and exhaustion) in the study, most likely attributed to the cognitive focus of the SCoRS assessment tool and the secondary nature of this study (i.e., burden was spontaneously reported by caregivers in the primary study). Fourth, results from primary and secondary caregivers were combined, and secondary analyses could not link specific primary or secondary caregivers to specific patients, thereby also not allowing to link caregiver burden to the reported cognitive impairment of the patient. Lastly, the study was conducted within the United States, and its findings may not be generalisable to countries with different social welfare and healthcare systems, where caregiver burden may be different, families (primary caregivers) may be driven by different social norms, or there are differences in secondary caregiver qualifications or community services. Future research should aim to explore caregiver burden of CIAS on a global and regional scale, and separately for primary and secondary caregivers as well as relate the caregiver burden to their assessment of the cognitive burden of the patient to better understand the full extent of the impact of patient cognitive impairment on caregivers.

In conclusion, this qualitative, secondary, thematic analysis study of caregivers for people with schizophrenia indicated a high level of objective caregiver burden related to CIAS symptoms among both primary and secondary caregivers. The principal themes identified were caregiver difficulty (Theme 1) and caregiver necessity (Theme 2). The requirement for both primary and secondary caregivers to assume extra roles and responsibilities due to the impacts of CIAS symptoms was central to these themes. The SCoRS domains of memory, learning, attention, problem-solving, language and social cognition were identified as areas to target with new treatments in patients with schizophrenia to alleviate caregiver burden. Further research is required to investigate the subjective burden experienced by primary and secondary caregivers of patients with schizophrenia.

## Supplementary information

Supplementary methods

## Data Availability

Parexel was contracted by Boehringer Ingelheim to conduct the analyses, interpret the results, as well as write, review and revise the manuscript. To ensure independent interpretation of clinical study results and enable authors to fulfil their role and obligations under the ICMJE criteria, Boehringer Ingelheim grants all external authors access to relevant clinical study data. In adherence with the Boehringer Ingelheim Policy on Transparency and Publication of Clinical Study Data, scientific and medical researchers can request access to clinical study data, typically, 1 year after the approval has been granted by major Regulatory Authorities or after termination of the development programme. Researchers should use the https://vivli.org/ link to request access to study data and visit https://www.mystudywindow.com/msw/datasharing for further information.
