# Supplementary material for: Primary and secondary caregiver burden of cognitive impairment associated with schizophrenia: a qualitative study based on caregiver interviews
Source: Schizophrenia (Heidelb). 2025 Oct 17;11(1):127. doi: 10.1038/s41537-025-00675-1 (PMC12534598; doi:10.1038/s41537-025-00675-1)
Supplement: Supplementary file 1 — Supplementary methods [file 41537_2025_675_MOESM1_ESM.pdf]

Supplementary methods

Interview discussion guide for primary caregivers

The interview discussion guide was not designed for this secondary qualitative study but was designed for the SCoRS concept confirmation study[1]

1. Correll, C. U. et al. Concept confirmation of the Schizophrenia Cognition Rating Scale (SCoRS) among unpaid and professional caregivers. *Schizophrenia* (2025; Submitted, under review)

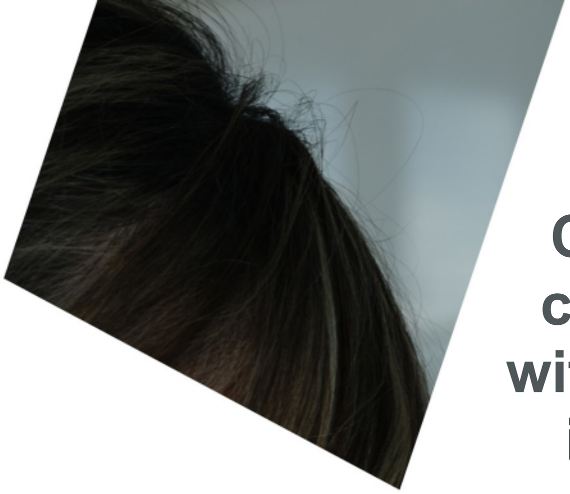

Concept elicitation on caregivers' experience with cognitive difficulties in schizophrenia and content confirmation of the Schizophrenia Cognition Rating Scale (SCoRS)

Interview Guide

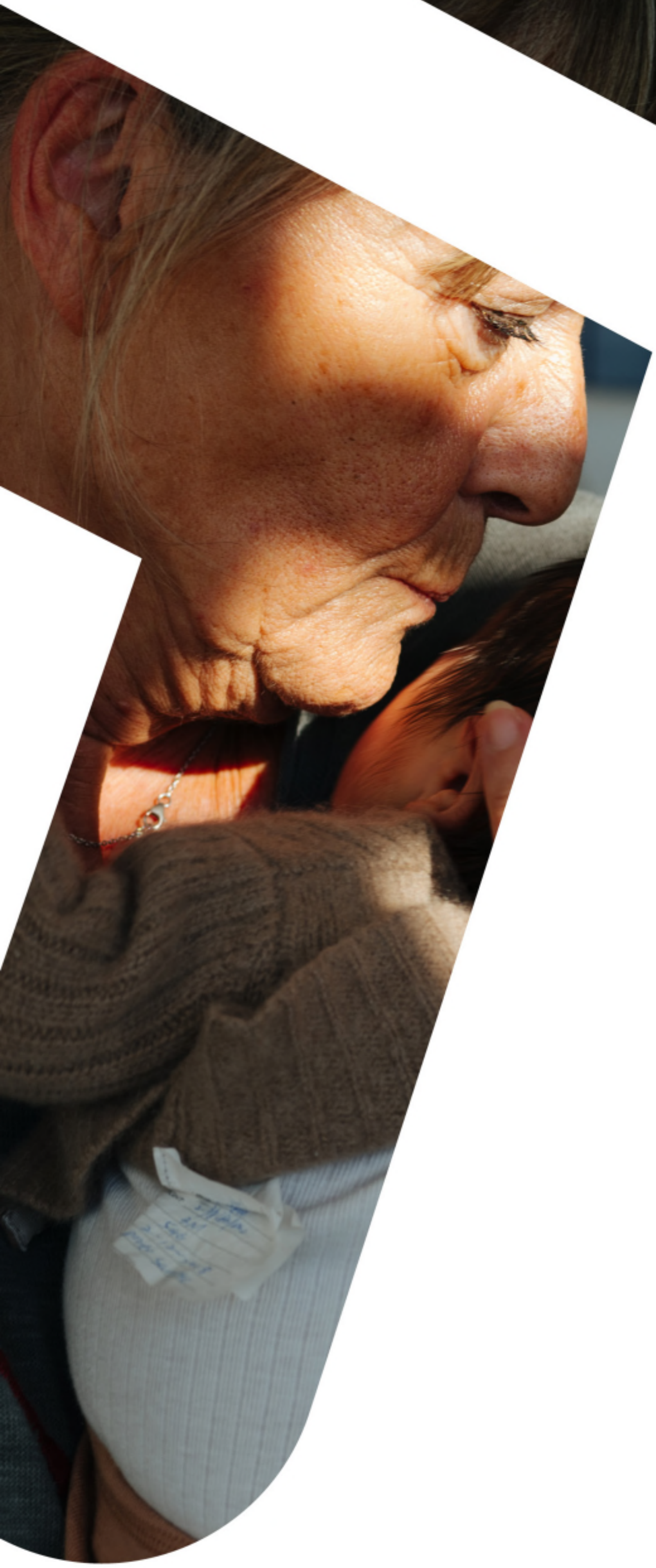

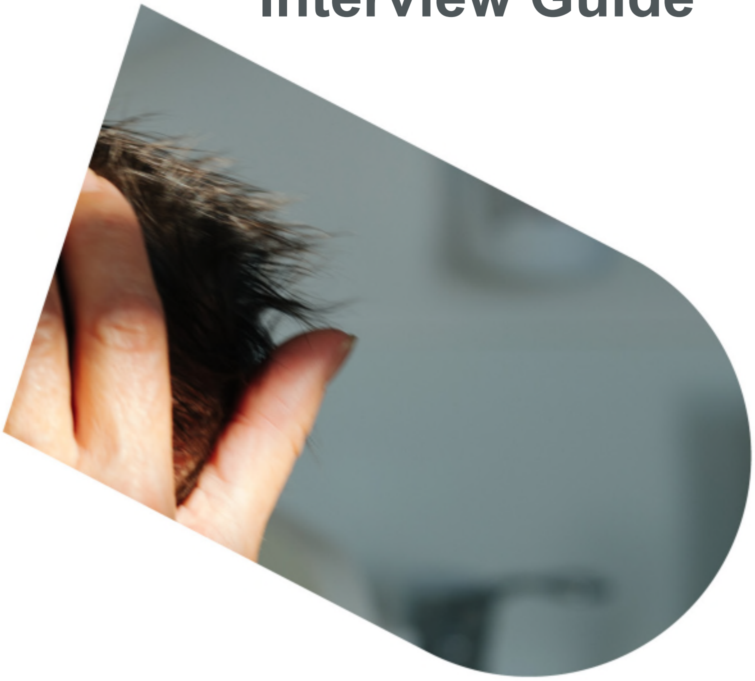

Version 1.7
29th April 2021

parexel

Introduction

Interviewer:

"Thank you for agreeing to participate in this interview. You have been asked to participate in this study because you have been identified as a caregiver of a person diagnosed with schizophrenia or someone who interacts with a person diagnosed with schizophrenia on a regular basis. The purpose of this interview is to learn more about cognitive problems people with schizophrenia might have. Cognition is a term referring to mental processes involved in knowledge and comprehension, for example, thinking, knowing, remembering, judging, and problem solving. Cognitive impairment, which refers to problems with thinking or mental functioning, is often associated with schizophrenia. Some examples of cognitive skills patients with schizophrenia may have problems with, are as follows:

**Learning and/or memory**	Remembering where they put something<br>Remembering what they were asked to buy at a store<br>Remembering a phone number just given to them<br>Remembering where they put something in the closet
**Attention**	Being able to follow a conversation<br>Being able to read a book or pay attention to a movie
**Problem solving**	Arriving on time when the bus schedule has changed
**Speed of processing**	Using a touch-screen computer, a vending machine display, or a new mobile phone<br>Making the correct change when using cash to pay for something

This interview consists of 3 parts: in the first part, I will ask you a few basic questions about yourself, in the second part, we will talk about your experiences with cognitive processes in people with schizophrenia like the person that you care for or frequently interact with; and in the third part, I will ask your opinion on a questionnaire called the Schizophrenia Cognition Rating Scale (SCoRS). The interview will last about 90 minutes.

As you know from being close to someone with schizophrenia or interacting frequently with patients with schizophrenia, there are many symptoms that they may experience such as delusions, hallucinations, difficult behaviors and so forth. But in this interview, we will only focus on difficulties around cognitive or mental processes, such as issues with memory, learning, and problem solving as mentioned above. The reason we are interested in these cognitive processes is that we are working with a pharmaceutical company called Boehringer Ingelheim that is developing a new medication that can potentially improve these cognitive difficulties in patients with schizophrenia. This pharmaceutical company is looking to use a

questionnaire called the Schizophrenia Cognition Rating Scale (SCoRS) to evaluate the benefit of this new medication. The SCoRS was developed in 2001 by Dr Richard Keefe at the Duke University Medical Center. The SCoRS is being used in a number of clinical and research studies, however, the Food and Drug Administration (FDA) will need to know if the SCoRS is relevant to these cognitive processes we mentioned to you. So that is why we want to talk to people who know patients with schizophrenia well, because they e.g. care for someone with schizophrenia or frequently interact with patients with schizophrenia.

With your permission, I will be audio-recording this interview. Please speak loudly and clearly so that all your comments can be captured on the audio recording. These audio recordings will be turned into a written document so that we can review the information later. Please note that we will not share the audio recordings with anyone outside of the research team. We will remove your personal information as well as that of the person with schizophrenia that you care for from the written document so that no information that could identify you personally or other people will be included in the final written document. You will be assigned an identification number in order to protect your confidentiality. We can share the written document with you, if you want to receive a copy. We will share the written document with all identifiable information removed, as well as the results of this study with Boehringer Ingelheim, the pharmaceutical company that sponsors this research and the FDA.

Do I have your permission to audio record today's interview?

□ Yes → Continue below

□ No → If no, inform the participant that they will not be able to participate if they do not want to be audio-recorded. If they still do not want to be recorded, end the interview.

After the interview, we will reimburse you via a bank transfer or an electronic Visa or Mastercard using Rybbon as a thank you for your participation. It is up to you to choose which payment method you prefer.

All information that you provide will be used for the purpose of this research project only. As mentioned earlier, no information that could identify you or the person with schizophrenia that you care for will be included in any reports or publications that result from this research. You do not have to answer any questions that make you feel uncomfortable or that you do not want to answer. We are interested in your thoughts and opinions. There are no right or wrong answers.

We are required to pass on to our sponsor details of adverse events/product complaints relating to their products if these are mentioned during the course of this study. Although what

you will say, of course will be treated in confidence, should you raise an adverse event or product complaint in a specific patient, or group of patients, we will need to report this event, even if it has already been reported by you directly to the company, to the doctor, or to the appropriate regulatory authorities. In case of an adverse event or product complaint, you will be asked whether you are willing to waive the confidentiality given to you specifically in relation to adverse event/product complaint.

Everything you say during the course of this discussion will continue to remain confidential, and you will still have the option to remain anonymous if you wish to do so.

Are you happy to participate in the discussion on this basis?

□ Yes → Continue below

□ No → If no, do not continue.

Do you have any questions about today's interview before we begin?"

Part 1: Informant questions/participation confirmation

Interviewer: "Before we get started, I have a few questions about yourself and your background."

1. How old are you?

   Instruction to interviewer: if a participant reports that he/she is 18, ask for a date of birth to judge if the participant is 18 on the day of screening. If participant is younger than 18 on the day of screening, end the interview.

2. What is your gender?

3. Which US state are you currently living in?

4. What is your highest level of education?

- High school (no degree) or less
- High school graduate (or equivalent)

- Some college (no degree)
- Associate degree
- Bachelor's degree
- Master's degree
- Doctoral degree
- Other (please specify):______________________

5. What is your relationship to {the patient}? (E.g. spouse/partner, relative (parent, child, sibling) or friend)?

6. How long have you been caring for/interacting with the person diagnosed with schizophrenia on a regular basis (E.g. 1 year, 1month, 1 week)?

7. How much contact have you had with {the patient} during the past two weeks (E.g. 2 hours per week, 4 hours within two weeks, 30 minutes a day each day)?

   Instruction to interviewer: if a participant interacts with the patient less than a minimum of 1 hour per week (and preferably, at least 2 times a week) and none of the interactions are in person (at least one interaction per week MUST be in person), end the interview.

8. Is the patient you currently care for an inpatient, outpatient or is in home care settings?
9. In the past year, has the patient been hospitalized for schizophrenia?

   If 'YES':
   a. How many times has the patient been hospitalized for schizophrenia?
   b. How many days/weeks in the past year did the patient spend in the hospital in total because of schizophrenia?

10. Does the patient currently take any antipsychotic medications for their schizophrenia?

    If 'YES'
    a. What are the medications the patient currently takes?

    Instruction to the interviewer: please explain what antipsychotic medication is in simple words in case the participant doesn't know. E.g. antipsychotic medications are a type of

medications to treat symptoms of hallucinations or delusions mostly in patients with schizophrenia.

11. Are you familiar with the SCoRS or have you been asked to participate in a SCoRS (Schizophrenia Cognition Rating Scale) interview prior to this study?

12. Are you capable (e.g. have sufficient hearing and vision etc.) to take part in 1 hour interview?

    Instruction to interviewer: if a participant responses YES to the hearing or vision problems, please check with the participant if they still can hear you well and understand what you are saying, and if they can see you and see the SCoRS. If you identify problems that prevent the participant to provide reliable answers, end the interview.

13. Are you able to read, write and speak in English to participate in an interview?

    Instruction to interviewer: if a participant is unable to read, write or speak in English, end the interview.

14. Do you agree to take part in this study?

    Instruction to interviewer: if a participant says NO to this question, end the interview.

Interview Part 2: Concept elicitation

Interviewer: "Based on your experience from caring for or interacting with a person with schizophrenia, you know that there are many different symptoms patients can experience. They may experience symptoms such as delusions, hallucinations, difficult behaviors and so forth. Today, however, we are going to focus only on difficulties related to their cognitive functioning in their daily life; this means we are interested in any issues around memory, remembering things, paying attention, learning, making decision, quick thinking, planning, ability to understand something or ability to concentrate on something,

1. "Please think about the person with schizophrenia that you care for or know well and describe the issues you have observed relating to their cognitive functioning in general or

on a normal day. Can you walk me through this experience and what problems you have observed?" What stands out to you as being particularly important or bothersome related to these issues?

Instruction to interviewer: After the subject has finished spontaneously describing cognitive difficulties as well as identified what they find particularly bothersome, follow up with the probing questions below.

Probing questions (probing as needed):

[MEMORY]
People with schizophrenia may also have difficulties related to memory such as:

- remembering names of people they know or have met (e.g. roommate, nurse, doctor, family & friends, etc.)
- remembering how to get to places (e.g. restroom, own room, friend's house, etc.)
- remembering where they put things (e.g. clothes, glasses, things, cigarettes, etc.)
  remembering tasks/chores they need to do (e.g. household chores, appointments)

Have you observed any of these issues? Can you walk me through this experience and what problems you have observed? What stands out to you as being particularly important or bothersome related to these memory issues?

[LEARNING]
People with schizophrenia may also have learning difficulties such as:

- learning how to use new gadgets and equipment (e.g. computers, washer, microwave, phone, remote, etc.)
- learning new things (e.g. new words, new ways of doing things, new schedules, etc.)

Have you observed any of these issues? Can you walk me through this experience and what problems you have observed? What stands out to you as being particularly important or bothersome related to these learning issues?

[ATTENTION]
Some individuals with schizophrenia may also have attention difficulties such as:

- following a TV show (e.g. favorite show, news, etc.)

- concentrating well enough to read a newspaper or a book (e.g., reading same sentence or page over and over)
- staying focused (e.g., daydream, trouble paying attention to someone talking, etc.)

Have you observed any of these issues? Can you walk me through this experience and what problems you have observed? What stands out to you as being particularly important or bothersome related to these attention issues?

[WORKING MEMORY]
Some individuals with schizophrenia may also struggle with:

- remembering what they were going to say (e.g., forgetting words, stopping mid-sentence)
- remembering information and/or instructions recently given to them (e.g., telephone numbers, directions, names, etc.)

Have you observed any of these issues? Can you walk me through this experience and what problems you have observed? What stands out to you as being particularly important or bothersome related to these memory issues?

[PROBLEM SOLVING]
People with schizophrenia may also have difficulties with some everyday tasks e.g.

- completing a familiar task (e.g., cooking, driving, showering, getting dressed, etc.)
- keeping track of money (e.g., managing bills, counting change, etc.)
- handling changes in daily routine (e.g., appointments, special visits, group therapy, etc.)

Have you observed any of these issues? Can you walk me through this experience and what problems you have observed? What stands out to you as being particularly important or bothersome related to these issues?

[PROCESSING/MOTOR SPEED]
People with schizophrenia may also have difficulties with:

- speaking as fast as they would like (e.g., slow speech, pauses)
- doing things quickly (e.g., writing, lighting a cigarette, etc.)

Have you observed any of these issues? Can you walk me through this experience and what problems you have observed? What stands out to you as being particularly important or bothersome related to these issues?

[COMMUNICATION/SOCIAL COGNITION] (Understanding language and social situations)
Some individuals with schizophrenia may also struggle with:

- understanding how other people feel about things (e.g., misunderstanding people's emotions by their facial expression or tone of their voice)
- following conversations in a group
- understanding what people mean when they are talking to them (e.g., feeling confused by what someone says)

Have you observed any of these issues? Can you walk me through this experience and what problems you have observed? What stands out to you as being particularly important or bothersome related to these issues?

[LANGUAGE] (Active language production)
Some individuals with schizophrenia may also experience difficulties with:

- keeping their words from being jumbled together (e.g. words get mixed up or "run together")
- participating in a group conversation

Have you observed any of these issues? Can you walk me through this experience and what problems you have observed? What stands out to you as being particularly important or bothersome related to these issues?

Interview Part 3: SCoRS - Cognitive Debriefing

Interviewer: “Thank you for your answers - they are really helpful. Now we will move on to the third part of our interview, during which we will review the questionnaire I mentioned earlier called the Schizophrenia Cognition Rating Scale or SCoRS. This questionnaire is used as an interview in clinical studies to collect information about cognition-related difficulties of patients with schizophrenia from people who know the patients well. Therefore, we want to find out whether this questionnaire can be easily understood. Please note that some questions may seem repetitive, but we want to capture all your thoughts about the questionnaire. First, I will ask you to listen to the questionnaire instructions and to share your thoughts with me. Then, we will go through each question of the questionnaire and I will ask you to talk me through your thoughts. This is what we call a think aloud exercise. Do you have any questions about what we are going to do before we start?”

Part 3A: SCoRS instruction clarity

<u>Interviewer</u>: "Please listen carefully to the instructions for the questionnaire that I will read to you. After that, I will ask you a few questions."

<u>Instructions to interviewer</u>: Please read the bold text in the box below that is intended to be read to the informant.

<u>Informant</u> Instructions:

Please read the following **bolded** text, <u>verbatim</u>, before beginning the informant interview on the next page.

Today I am going to ask you questions about the level of difficulty {the patient, your son/daughter, your roommate…etc.*} may experience with certain tasks. The ratings for each question are none, mild, moderate, and severe. He/she may have 'no' difficulty or maybe they have 'severe' difficulty with the task. With other tasks you may feel that they have only 'mild' or 'moderate' difficulty. Do your best to answer each question as honestly as you can. You should think about the amount of difficulty that {the patient} has experienced in the past two weeks. If you are not sure what I am asking, stop me and I will explain it to you.

1. "In your own words, what are these instructions telling you to do?"

2. "How easy or difficult are the instructions to understand?"

   <u>Instructions to interviewer</u>: Follow up with probes
   - *If easy:* "What makes them easy to understand?
   - *If difficult:* "What makes them difficult to understand?

3. "How easy or difficult is it to think about the past 2 weeks when thinking about the cognitive difficulties of the {patient}?"

Part 3B: SCoRS item clarity

<u>Interviewer</u>: "Now we will start with the questionnaire – the questionnaire has 20 questions. As you saw in the instructions, I am going to ask you questions about the level of difficulty {the patient, your son/daughter, your roommate…etc.} may experience with certain tasks. The responses for each question are none, mild, moderate, and severe. He/she may have 'no' difficulty or maybe they have 'severe' difficulty with the task. With other tasks you may feel that they have only 'mild' or 'moderate' difficulty. Do your best to answer each question as honestly as you can. You should think about the amount of difficulty that {the patient} has

experienced in the past two weeks. If you are not sure what I am asking, please stop me and I will explain it to you. Do you have any questions?”

Instruction to the interviewer: Make sure to answer all of the informant’s questions before proceeding.

Item 1

“The first question of the ScoRS questionnaire is the following: does the {patient} have difficulty remembering names of people they know or meet? For example, roommate, nurse, doctor, family, and friends?”

4. “In your own words, what does this question ask about?”

Instruction to interviewer: Follow up with probes as needed (e.g., “What is it asking you to do?”)

5. Now we would like to talk about response options for this question. The response options are:

None: No difficulties
Mild: Remembers most names of people that he/she knows but not all of the people he/she has just met
Moderate: Forgets many names of people he/she knows and all of the names of people he/she has just met
Severe: Forgets all or almost all names of people he/she knows and meets

’’How easy or difficult is it to pick a response?’’

“Now we are moving on to the next question.”

Item 2

“Question 2 of the SCoRS is the following: does the {patient} you care for have difficulty remembering how to get places? For example, how to get to the restroom, their own room, a friend’s house?”

6. “In your own words, what does this question ask about?”

Instruction to interviewer: Follow up with probes as needed (e.g., "What is it asking you to do? How helpful are the provided examples?")

7. Now we would like to talk about response options for this question. The response options are:

None: No difficulties
Mild: Forgets infrequently
Moderate: Is only able to get to frequently visited places
Severe: Unable to get anyplace without assistance because difficulties with memory

''How easy or difficult is it to pick a response?''

"Now we are moving on to the next question."

Item 3

"Question 3 of the SCoRS is the following: does the {patient} you care for have difficulty following a TV show? For example, a favorite show or the news."

8. "In your own words, what does this question ask about?"

Instruction to interviewer: Follow up with probes as needed (e.g., "What is it asking you to do? How helpful are the provided examples?")

9. Now we would like to talk about response options for this question. The response options are:

None: No difficulties
Mild: Can only follow a short movie or news show
Moderate: Can only follow a light, 30 minutes show (i.e. sitcom)
Severe: Unable to follow a TV show for any period of time

''How easy or difficult is it to pick a response?''

"Now we are moving on to the next question."

Item 4

"Question 4 of the SCoRS is the following: does the {patient} you care for have difficulty remembering where they put things? For example, where they put clothes, the newspaper, or cigarettes?"

10. "In your own words, what does this question ask about?"

Instruction to interviewer: Follow up with probes as needed (e.g., "What is it asking you to do? How helpful are the provided examples?")

11. Now we would like to talk about response options for this question. The response options are:

None: No difficulties
Mild: Rare instances of forgetfulness
Moderate: Frequent instances of forgetfulness
Severe: Very frequent instances of forgetfulness or forgetting items of great importance

''How easy or difficult is it to pick a response?''

"Now we are moving on to the next question."

Item 5

"Question 5 of the SCoRS is the following: does the {patient} you care for have difficulty remembering their chores and responsibilities? For example, household chores or appointments?"

12. "In your own words, what does this question ask about?"

Instruction to interviewer: Follow up with probes as needed (e.g., "What is it asking you to do? How helpful are the provided examples?")

13. Now we would like to talk about response options for this question. The response options are:

None: No difficulties
Mild: Infrequently forgets
Moderate: Forgets only those things that do not occur everyday
Severe: Forgets all or almost all of his/her responsibilities

‘‘How easy or difficult is it to pick a response?’’

“Now we are moving on to the next question.”

Item 6

“Question 6 of the SCoRS is the following: does the {patient} you care for have difficulty learning how to use new gadgets and equipment? For example, computer, washer, microwave, phone, remote, DVR?”

14. “In your own words, what does this question ask about?”

Instruction to interviewer: Follow up with probes as needed (e.g., “What is it asking you to do? How helpful are the provided examples?”)

15. Now we would like to talk about response options for this question. The response options are:

None: No difficulties
Mild: Takes longer to learn than most, but can usually do it
Moderate: Takes longer and needs to be taught; can not learn some things
Severe: Unable to learn how to use new gadgets and equipment

‘‘How easy or difficult is it to pick a response?’’

“Now we are moving on to the next question.”

Item 7

“Question 7 of the SCoRS is the following: does the {patient} you care for have difficulty remembering information and/or instructions recently given to them? For example, telephone numbers, directions, names?”

16. “In your own words, what does this question ask about?”

Instruction to interviewer: Follow up with probes as needed (e.g., "What is it asking you to do? How helpful are the provided examples?")

17. Now we would like to talk about response options for this question. The response options are:

None: No difficulties
Mild: Rarely has difficulty remembering information
Moderate: Frequently forgets information given
Severe: Almost always forgets information

''How easy or difficult is it to pick a response?''

"Now we are moving on to the next question."

Item 8

"Question 8 of the SCoRS is the following: does the {patient} you care for have difficulty remembering what they were going to say? For example, forgetting words, or stopping mid-sentence?"

18. "In your own words, what does this question ask about?"

Instruction to interviewer: Follow up with probes as needed (e.g., "What is it asking you to do? How helpful are the provided examples?")

19. Now we would like to talk about response options for this question. The response options are:

None: No difficulties
Mild: Rare instances of forgetfulness when speaking
Moderate: Frequent instances of forgetfulness when speaking
Severe: Frequency of forgetfulness makes communication very difficult

''How easy or difficult is it to pick a response?''

"Now we are moving on to the next question."

Item 9

"Question 9 of the SCoRS is the following: does the {patient} you care for have difficulty keeping track of their money? For example, managing bills or counting change?"

20. "In your own words, what does this question ask about?"

Instruction to interviewer: Follow up with probes as needed (e.g., "What is it asking you to do? How helpful are the provided examples?")

21. Now we would like to talk about response options for this question. The response options are:

None: No difficulties
Mild: Some difficulty but can usually do it
Moderate: Significant difficulty either with counting change or paying bills
Severe: Unable to keep track of his/her money because of cognitive difficulties

''How easy or difficult is it to pick a response?''

"Now we are moving on to the next question."

Item 10

"Question 10 of the SCoRS is the following: does the {patient} you care for have difficulty keeping their words from being jumbled together? For example, words get mixed up or "run together"?"

22. "In your own words, what does this question ask about?"

Instruction to interviewer: Follow up with probes as needed (e.g., "What is it asking you to do? How helpful are the provided examples?")

23. Now we would like to talk about response options for this question. The response options are:

None: No difficulties
Mild: Sometimes will jumble words but it's rare
Moderate: Can have a conversation but jumbles words frequently

Severe: Unable to have a conversation due to jumbled words

''How easy or difficult is it to pick a response?''

"Now we are moving on to the next question."

Item 11

"Question 11 of the SCoRS is the following: does the {patient} you care for have difficulty concentrating well enough to read a newspaper or a book? For example, reading the same sentence or the same page over and over?"

24. "In your own words, what does this question ask about?"

Instruction to interviewer: Follow up with probes as needed (e.g., "What is it asking you to do? How helpful are the provided examples?")

25. Now we would like to talk about response options for this question. The response options are:

None: No difficulties
Mild: Can concentrate except for rare occasions
Moderate: Can concentrate on short and easy to understand materials
Severe: Unable to read even the simplest materials due to concentration problems

''How easy or difficult is it to pick a response?''

"Now we are moving on to the next question."

Item 12

"Question 12 of the SCoRS is the following: does the {patient} you care for have difficulty with familiar tasks? For example cooking, driving, showering, getting dressed?"

26. "In your own words, what does this question ask about?"

Instruction to interviewer: Follow up with probes as needed (e.g., "What is it asking you to do? How helpful are the provided examples?")

27. Now we would like to talk about response options for this question. The response options are:

None: No difficulties
Mild: Rarely has difficulty completing the task
Moderate: Frequently needs verbal assistance to complete the task
Severe: Needs physical assistance to do these tasks due to cognitive difficulties

''How easy or difficult is it to pick a response?''

"Now we are moving on to the next question."

Item 13

"Question 13 of the SCoRS is the following: does the {patient} you care for have difficulty staying focused? For example, daydreaming, trouble paying attention to someone talking?"

28. "In your own words, what does this question ask about?"

Instruction to interviewer: Follow up with probes as needed (e.g., "What is it asking you to do? How helpful are the provided examples?")

29. Now we would like to talk about response options for this question. The response options are:

None: No difficulties
Mild: Sometimes unable to stay focused
Moderate: Frequently unable to stay focused
Severe: Almost always unable to stay focused

''How easy or difficult is it to pick a response?''

"Now we are moving on to the next question."

Item 14

"Question 14 of the SCoRS is the following: does the {patient} you care for have difficulty learning new things? For example, new words, new ways of doing things, new schedules?"

30. "In your own words, what does this question ask about?"

Instruction to interviewer: Follow up with probes as needed (e.g., "What is it asking you to do? How helpful are the provided examples?")

31. Now we would like to talk about response options for this question. The response options are:

None: No difficulties
Mild: Takes longer to learn than most, but can usually do it
Moderate: Takes longer and needs special attention
Severe: Unable to learn almost all new things

''How easy or difficult is it to pick a response?''

"Now we are moving on to the next question."

Item 15

"Question 15 of the SCoRS is the following: does the {patient} you care for have difficulty speaking as fast as they would like? For example, slow speech or pauses?"

32. "In your own words, what does this question ask about?"

Instruction to interviewer: Follow up with probes as needed (e.g., "What is it asking you to do? How helpful are the provided examples?")

33. Now we would like to talk about response options for this question. The response options are:

None: No difficulties
Mild: Rarely speaks slowly because of cognitive difficulties
Moderate: Often speaks slowly because of cognitive difficulties

Severe: Ability to converse is jeopardized because of cognitive difficulties

''How easy or difficult is it to pick a response?''

"Now we are moving on to the next question."

Item 16

"Question 16 of the SCoRS is the following: does the {patient} you care for have difficulty doing things quickly? For example, writing or lighting a cigarette?"

34. "In your own words, what does this question ask about?"

Instruction to interviewer: Follow up with probes as needed (e.g., "What is it asking you to do? How helpful are the provided examples?")

35. Now we would like to talk about response options for this question. The response options are:

None: No difficulties
Mild: Slightly slower than normal pace
Moderate: Significantly slower; may need prompting to do things quickly
Severe: Unable to get things done because time runs out

''How easy or difficult is it to pick a response?''

"Now we are moving on to the next question."

Item 17

"Question 17 of the SCoRS is the following: does the {patient} you care for have difficulty handling changes in their daily routine? For example, appointments, special visits, or group therapy?"

36. "In your own words, what does this question ask about?"

Instruction to interviewer: Follow up with probes as needed (e.g., "What is it asking you to do? How helpful are the provided examples?")

37. Now we would like to talk about response options for this question. The response options are:

None: No difficulties
Mild: Can adjust with considerable effort
Moderate: Will eventually adjust with assistance
Severe: Changes in the daily routine are impossible

‘‘How easy or difficult is it to pick a response?’’

“Now we are moving on to the next question.”

Item 18

“Question 18 of the SCoRS is the following: does the {patient} you care for have difficulty understanding what people mean when they are talking to them? For example, feeling confused by what someone says?”

38. “In your own words, what does this question ask about?”

Instruction to interviewer: Follow up with probes as needed (e.g., “What is it asking you to do? How helpful are the provided examples?”)

39. Now we would like to talk about response options for this question. The response options are:

None: No difficulties
Mild: Some difficulty understanding what people mean
Moderate: Often has difficulty understanding what people mean
Severe: Frequently unable to understand what people mean

‘‘How easy or difficult is it to pick a response?’’

“Now we are moving on to the next question.”

Item 19

"Question 19 of the SCoRS is the following: does the {patient} you care for have difficulty understanding how other people feel about things? For example, misunderstanding people's emotions by their facial expressions or tone of their voice?"

40. "In your own words, what does this question ask about?"

Instruction to interviewer: Follow up with probes as needed (e.g., "What is it asking you to do? How helpful are the provided examples?")

41. Now we would like to talk about response options for this question. The response options are:

None: No difficulties
Mild: Rarely has difficulty understanding how people feel
Moderate: Often has difficulty understanding how people feel
Severe: Very frequent instances of difficulty understanding how people feel

''How easy or difficult is it to pick a response?''

"Now we are moving on to the next question."

Item 20

"Question 20 of the SCoRS is the following: does the {patient} you care for have difficulty following conversations in a group? For example, participation, able to follow a conversation?"

42. "In your own words, what does this question ask about?"

Instruction to interviewer: Follow up with probes as needed (e.g., "What is it asking you to do? How helpful are the provided examples?")

43. Now we would like to talk about response options for this question. The response options are:

None: No difficulties
Mild: Few difficulties following conversations in a group
Moderate: Often unable to follow conversations in a group
Severe: Frequently unable to follow conversations in a group and communication in that setting is difficult or impossible

''How easy or difficult is it to pick a response?''

"Now we are moving on to some more instructions."

44. We would also like to know the degree of severity of cognitive impairment of the person with schizophrenia that you care for.

Please choose the response below that best describes the degree of severity of the person's cognitive impairment in the past two weeks.

 None

 Mild

 Moderate

 Severe

45. Finally, we would like to know whether you think there are any important symptoms or impacts of cognitive impairments that should be added or removed from the SCoRS questionnaire?

Instruction to the interviewer: If the respondent says 'Yes' to adding to removing items from SCoRS, and the respondent to explain why they think items should be added or removed.

Part 3C: SCoRS follow up instruction clarity & informants rating

Instruction to interviewer: Read the bolded text in the instruction box below. Prompt/probe as necessary to help establish and clarify proper timeframe.

Interviewer: "Now I'm going to read the next instructions in the SCoRS questionnaire to you. This questionnaire will be used in clinical trials to interview people like you, who care for or work with schizophrenia patients and know them well, in order to assess how much the patient's cognitive functioning has improved during the clinical trial. Imagine, your patient/the person you are caring for, has just completed his/her participation in a clinical trial and had tested a new mediation. Can you please listen to the instructions I am going to read now? Once I'm done reading the instructions, I will ask you a few questions."

Informant Instructions:
Please read the following **bolded** text, verbatim. Circle answer.
I want you to think about how difficult it was for {the patient, your son/daughter, your roommate…etc.*} to perform these tasks since they started taking the study medication. [Prompt/probe as necessary to help establish and clarify proper timeframe.]. Now I want you to think about how difficult it is for him/her to do the things we just discussed today. Rate how their level of difficulty with these tasks has changed. I want you to do this using a scale of 1-7 with 1 being "much worse", 4 being "the same", and 7 being "much better".

46. "In your own words, what are these instructions telling people to do?"

47. "How easy or difficult are the instructions to understand?"

Instructions to interviewer: Follow up with probes

- *If easy:* "What makes them easy to understand?
- *If difficult:* "What makes them difficult to understand?

Interviewer: "Now we will look at the scale."

"Please look at the scale and I will ask you a few questions about it."

much worse	INFORMANT RATES	much improved

1-----------------2-----------------3-----------------4-----------------5-----------------6---------------7

1	2	3	4	5	6	7
much worse	moderately. worse	minimally worse	no change	minimally. improved	moderately improved	much improved

48. "In your own words, what does "much improved" mean to you?"

49. "In your own words, what does "moderately improved" mean to you?"

50. "In your own words, what does "minimally improved" mean to you?"

51. "In your own words, what does "no change" mean to you?"

52. "In your own words, what does "minimally worse" mean to you?"

53. "In your own words, what does "moderately worse" mean to you?"

54. "In your own words, what does "much worse" mean to you?"

55. "How easy or difficult is it to pick an answer along the scale?"

Instructions to interviewer: Follow up with probes

- *If easy:* "What makes it easy?
- *If difficult:* "What makes it difficult?

"This was the final question. Thank you very much for participating in this study. We very much appreciate your answers. Do you have any final comments that you would like to share before we end the interview?"

Supplementary methods

Interview discussion guide for secondary caregivers

The interview discussion guide was not designed for this secondary qualitative study but was designed for the SCoRS concept confirmation study[1]

1. Correll, C. U. et al. Concept confirmation of the Schizophrenia Cognition Rating Scale (SCoRS) among unpaid and professional caregivers. *Schizophrenia* (2025; Submitted, under review)

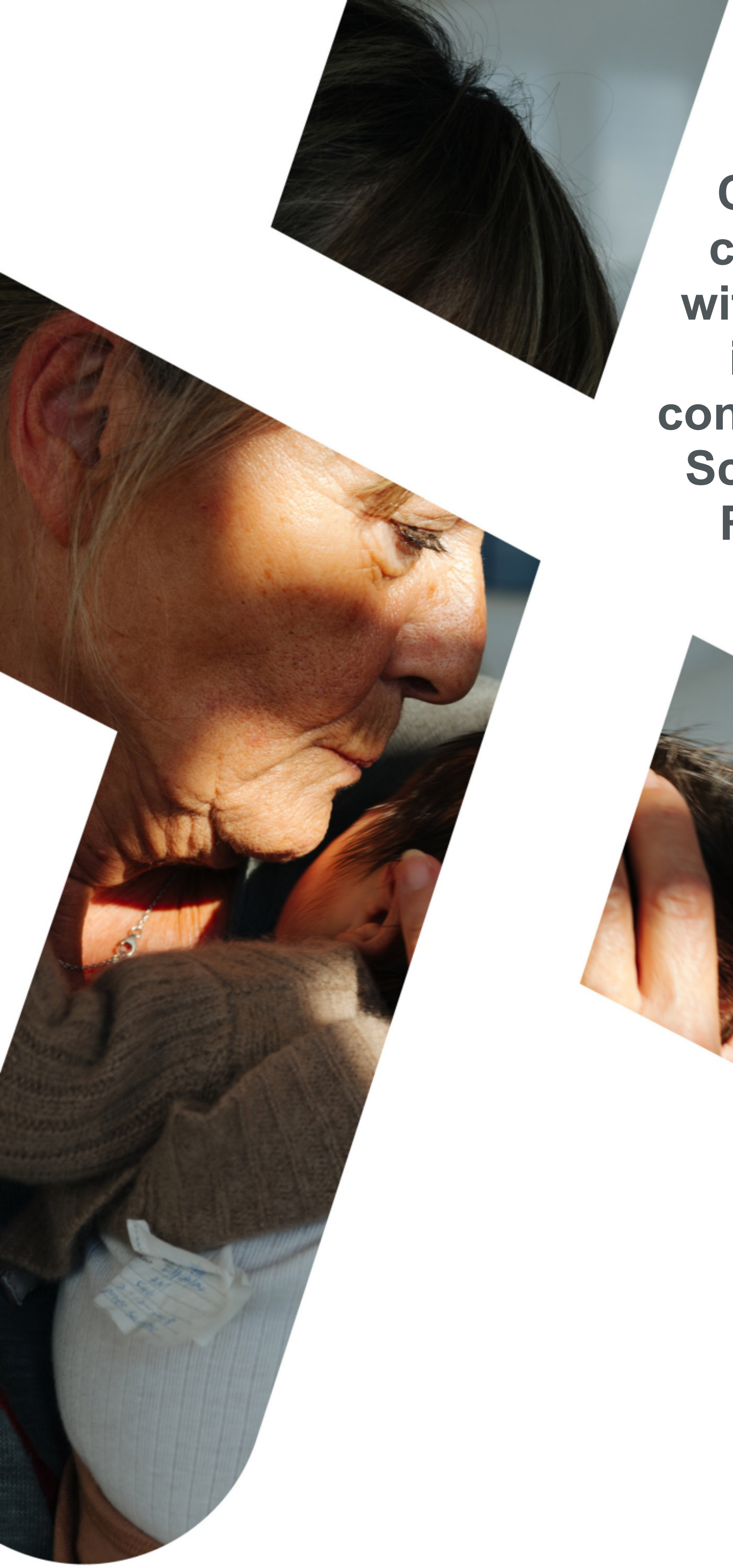

Concept elicitation on caregivers' experience with cognitive difficulties in schizophrenia and content confirmation of the Schizophrenia Cognition Rating Scale (SCoRS)

Interview Guide

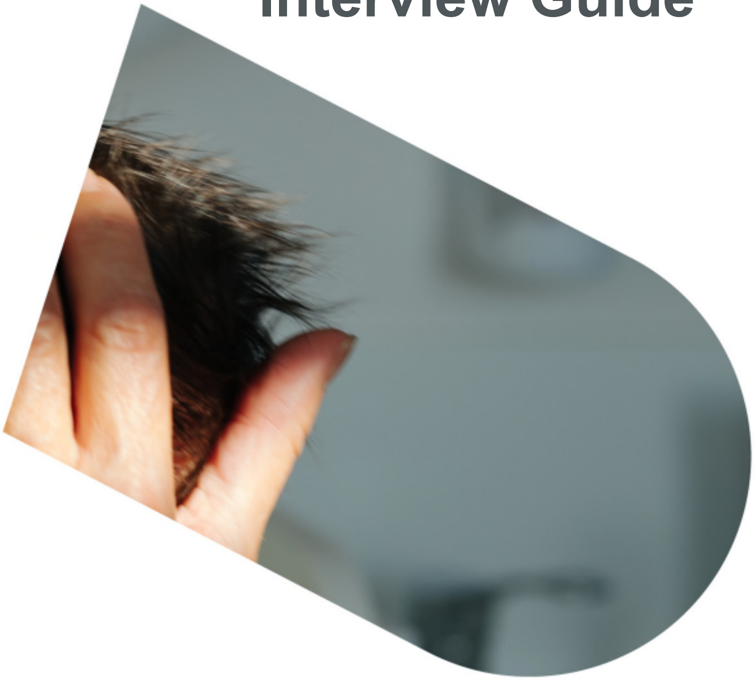

Version 1.7
29th April 2021

parexel

Introduction

Interviewer:

“Thank you for agreeing to participate in this interview. You have been asked to participate in this study because you have been identified as a caregiver of a person diagnosed with schizophrenia or someone who interacts with a person diagnosed with schizophrenia on a regular basis. The purpose of this interview is to learn more about cognitive problems people with schizophrenia might have. Cognition is a term referring to mental processes involved in knowledge and comprehension, for example, thinking, knowing, remembering, judging, and problem solving. Cognitive impairment, which refers to problems with thinking or mental functioning, is often associated with schizophrenia. Some examples of cognitive skills patients with schizophrenia may have problems with, are as follows:

**Learning and/or memory**	Remembering where they put something<br>Remembering what they were asked to buy at a store<br>Remembering a phone number just given to them<br>Remembering where they put something in the closet
**Attention**	Being able to follow a conversation<br>Being able to read a book or pay attention to a movie
**Problem solving**	Arriving on time when the bus schedule has changed
**Speed of processing**	Using a touch-screen computer, a vending machine display, or a new mobile phone<br>Making the correct change when using cash to pay for something

This interview consists of 3 parts: in the first part, I will ask you a few basic questions about yourself, in the second part, we will talk about your experiences with cognitive processes in people with schizophrenia like the person that you care for or frequently interact with; and in the third part, I will ask your opinion on a questionnaire called the Schizophrenia Cognition Rating Scale (SCoRS). The interview will last about 90 minutes.

As you know from being close to someone with schizophrenia or interacting frequently with patients with schizophrenia, there are many symptoms that they may experience such as delusions, hallucinations, difficult behaviors and so forth. But in this interview, we will only focus on difficulties around cognitive or mental processes, such as issues with memory, learning, and problem solving as mentioned above. The reason we are interested in these cognitive processes is that we are working with a pharmaceutical company called Boehringer Ingelheim that is developing a new medication that can potentially improve these cognitive difficulties in patients with schizophrenia. This pharmaceutical company is looking to use a

questionnaire called the Schizophrenia Cognition Rating Scale (SCoRS) to evaluate the benefit of this new medication. The SCoRS was developed in 2001 by Dr Richard Keefe at the Duke University Medical Center. The SCoRS is being used in a number of clinical and research studies, however, the Food and Drug Administration (FDA) will need to know if the SCoRS is relevant to these cognitive processes we mentioned to you. So that is why we want to talk to people who know patients with schizophrenia well, because they e.g. care for someone with schizophrenia or frequently interact with patients with schizophrenia.

With your permission, I will be audio-recording this interview. Please speak loudly and clearly so that all your comments can be captured on the audio recording. These audio recordings will be turned into a written document so that we can review the information later. Please note that we will not share the audio recordings with anyone outside of the research team. We will remove your personal information as well as that of the person with schizophrenia that you care for from the written document so that no information that could identify you personally or other people will be included in the final written document. You will be assigned an identification number in order to protect your confidentiality. We can share the written document with you, if you want to receive a copy. We will share the written document with all identifiable information removed, as well as the results of this study with Boehringer Ingelheim, the pharmaceutical company that sponsors this research and the FDA.

Do I have your permission to audio record today's interview?

□ Yes → Continue below

□ No → If no, inform the participant that they will not be able to participate if they do not want to be audio-recorded. If they still do not want to be recorded, end the interview.

After the interview, we will reimburse you via a bank transfer or an electronic Visa or Mastercard using Rybbon as a thank you for your participation. It is up to you to choose which payment method you prefer.

All information that you provide will be used for the purpose of this research project only. As mentioned earlier, no information that could identify you or the person with schizophrenia that you care for will be included in any reports or publications that result from this research. You do not have to answer any questions that make you feel uncomfortable or that you do not want to answer. We are interested in your thoughts and opinions. There are no right or wrong answers.

We are required to pass on to our sponsor details of adverse events/product complaints relating to their products if these are mentioned during the course of this study. Although what

you will say, of course will be treated in confidence, should you raise an adverse event or product complaint in a specific patient, or group of patients, we will need to report this event, even if it has already been reported by you directly to the company, to the doctor, or to the appropriate regulatory authorities. In case of an adverse event or product complaint, you will be asked whether you are willing to waive the confidentiality given to you specifically in relation to adverse event/product complaint.

Everything you say during the course of this discussion will continue to remain confidential, and you will still have the option to remain anonymous if you wish to do so.

Are you happy to participate in the discussion on this basis?

□ Yes → Continue below

□ No → If no, do not continue.

Do you have any questions about today's interview before we begin?"

Part 1: Informant questions/participation confirmation

Interviewer: "Before we get started, I have a few questions about yourself and your background."

1. How old are you?

   Instruction to interviewer: if a participant reports that he/she is 18, ask for a date of birth to judge if the participant is 18 on the day of screening. If participant is younger than 18 on the day of screening, end the interview.

2. What is your gender?

3. Which US state are you currently living in?

4. What is your highest level of education?
   - High school (no degree) or less
   - High school graduate (or equivalent)

- Some college (no degree)
- Associate degree
- Bachelor’s degree
- Master’s degree
- Doctoral degree
- Other (please specify):_____________________

5. What is the type of your professional relationship to the patient(s) (E.g. nurse, case manager, social worker)?

6. How long have you been working with individuals with schizophrenia (E.g. 1 year, 1month, 1 week)?

7. For this question, please consider the patient with schizophrenia you <u>currently</u> spent the most time with, in case you provide care for more than one patient. How much contact have you had with the patient you spent the most time with during the past two weeks (E.g. 2 hours per week, 4 hours within two weeks, 30 minutes a day each day)?

    Instruction to interviewer: if a participant interacts with the patient less than a minimum of 1 hour per week (and preferably, at least 2 times a week) and none of the interactions are in person (at least one interaction per week MUST be in person), end the interview.

8. Do you work with this patient in inpatient, outpatient, or home care settings?

9. How long have you been working with this patient (E.g. 1 year, 1month, 1 week)?

10. In the past year, has the patient been hospitalized for schizophrenia?

    If ‘YES’:
    a. How many times has the patient been hospitalized for schizophrenia?
    b. How many days/weeks in the past year did the patient spend in the hospital in total because of schizophrenia?

11. Does the patient currently take any antipsychotic medications for their schizophrenia?

If 'YES'

a. What are the antipsychotic medications the patient currently takes?

Instruction the interviewer: please explain what antipsychotic medication is in simple words in case the participant doesn't know. E.g. antipsychotic medications are a type of medications to treat symptoms of hallucinations or delusions mostly in patients with schizophrenia.

12. Are you familiar with the SCoRS or have you been asked to participate in a SCoRS (Schizophrenia Cognition Rating Scale) interview prior to this study?

13. Are you capable (e.g. have sufficient hearing and vision) to take part in 1 hour interview?

Instruction to interviewer: if a participant responses YES to the hearing or vision problems, please check with the participant if they still can hear you well and understand what you are saying, and if they can see you and see the SCoRS. If you identify problems that prevent the participant to provide reliable answers, end the interview.

14. Are you able to read, write and speak in English to participate in an interview?

Instruction to interviewer: if a participant is unable to read, write or speak in English, end the interview.

15. Do you agree to take part in this study?

Instruction to interviewer: if a participant says NO to this question, end the interview.

Interview Part 2: Concept elicitation

Interviewer: "Based on your experience from caring for or interacting with a person with schizophrenia, you know that there are many different symptoms patients can experience. They may experience symptoms such as delusions, hallucinations, difficult behaviors and so forth. Today, however, we are going to focus only on difficulties related to their cognitive functioning in their daily life; this means we are interested in any issues around memory, remembering

things, paying attention, learning, making decision, quick thinking, planning, ability to understand something or ability to concentrate on something,

1. “Please think about the person with schizophrenia that you care for or know well and describe the issues you have observed relating to their cognitive functioning in general or on a normal day. Can you walk me through this experience and what problems you have observed?” What stands out to you as being particularly important or bothersome related to these issues?

Instruction to interviewer: After the subject has finished spontaneously describing cognitive difficulties as well as identified what they find particularly bothersome, follow up with the probing questions below.

Probing questions (probing as needed):

[MEMORY]
People with schizophrenia may also have difficulties related to memory such as:

- remembering names of people they know or have met (e.g. roommate, nurse, doctor, family & friends, etc.)
- remembering how to get to places (e.g. restroom, own room, friend’s house, etc.)
- remembering where they put things (e.g. clothes, glasses, things, cigarettes, etc.)
- remembering tasks/chores they need to do (e.g. household chores, appointments)

Have you observed any of these issues? Can you walk me through this experience and what problems you have observed? What stands out to you as being particularly important or bothersome related to these memory issues?

[LEARNING]
People with schizophrenia may also have learning difficulties such as:

- learning how to use new gadgets and equipment (e.g. computers, washer, microwave, phone, remote, etc.)
- learning new things (e.g. new words, new ways of doing things, new schedules, etc.)

Have you observed any of these issues? Can you walk me through this experience and what problems you have observed? What stands out to you as being particularly important or bothersome related to these learning issues?

[ATTENTION]
Some individuals with schizophrenia may also have attention difficulties such as:

- following a TV show (e.g. favorite show, news, etc.)
- concentrating well enough to read a newspaper or a book (e.g., reading same sentence or page over and over)
- staying focused (e.g., daydream, trouble paying attention to someone talking, etc.)

Have you observed any of these issues? Can you walk me through this experience and what problems you have observed? What stands out to you as being particularly important or bothersome related to these attention issues?

[WORKING MEMORY]
Some individuals with schizophrenia may also struggle with:

- remembering what they were going to say (e.g., forgetting words, stopping mid-sentence)
- remembering information and/or instructions recently given to them (e.g., telephone numbers, directions, names, etc.)

Have you observed any of these issues? Can you walk me through this experience and what problems you have observed? What stands out to you as being particularly important or bothersome related to these memory issues?

[PROBLEM SOLVING]
People with schizophrenia may also have difficulties with some everyday tasks e.g.

- completing a familiar task (e.g., cooking, driving, showering, getting dressed, etc.)
- keeping track of money (e.g., managing bills, counting change, etc.)
- handling changes in daily routine (e.g., appointments, special visits, group therapy, etc.)

Have you observed any of these issues? Can you walk me through this experience and what problems you have observed? What stands out to you as being particularly important or bothersome related to these issues?

[PROCESSING/MOTOR SPEED]
People with schizophrenia may also have difficulties with:

- speaking as fast as they would like (e.g., slow speech, pauses)
- doing things quickly (e.g., writing, lighting a cigarette, etc.)

Have you observed any of these issues? Can you walk me through this experience and what problems you have observed? What stands out to you as being particularly important or bothersome related to these issues?

[COMMUNICATION/SOCIAL COGNITION] (Understanding language and social situations)
Some individuals with schizophrenia may also struggle with:

- understanding how other people feel about things (e.g., misunderstanding people's emotions by their facial expression or tone of their voice)
- following conversations in a group
- understanding what people mean when they are talking to them (e.g., feeling confused by what someone says)

Have you observed any of these issues? Can you walk me through this experience and what problems you have observed? What stands out to you as being particularly important or bothersome related to these issues?

[LANGUAGE] (Active language production)
Some individuals with schizophrenia may also experience difficulties with:

- keeping their words from being jumbled together (e.g. words get mixed up or "run together")
- participating in a group conversation

Have you observed any of these issues? Can you walk me through this experience and what problems you have observed? What stands out to you as being particularly important or bothersome related to these issues?

Interview Part 3: SCoRS - Cognitive Debriefing

<u>Interviewer</u>: “Thank you for your answers - they are really helpful. Now we will move on to the third part of our interview, during which we will review the questionnaire I mentioned earlier called the Schizophrenia Cognition Rating Scale or SCoRS. This questionnaire is used as an interview in clinical studies to collect information about cognition-related difficulties of patients with schizophrenia from people who know the patients well. Therefore, we want to find out whether this questionnaire can be easily understood. Please note that some questions may seem repetitive, but we want to capture all your thoughts about the questionnaire. First, I will ask you to listen to the questionnaire instructions and to share your thoughts with me. Then, we will go through each question of the questionnaire and I will ask you to talk me through your thoughts. This is what we call a think aloud exercise. Do you have any questions about what we are going to do before we start?”

Part 3A: SCoRS instruction clarity

Interviewer: "Please listen carefully to the instructions for the questionnaire that I will read to you. After that, I will ask you a few questions."

Instructions to interviewer: Please read the bold text in the box below that is intended to be read to the informant.

Informant Instructions:

Please read the following **bolded** text, verbatim, before beginning the informant interview on the next page.

Today I am going to ask you questions about the level of difficulty {the patient, your son/daughter, your roommate…etc.*} may experience with certain tasks. The ratings for each question are none, mild, moderate, and severe. He/she may have 'no' difficulty or maybe they have 'severe' difficulty with the task. With other tasks you may feel that they have only 'mild' or 'moderate' difficulty. Do your best to answer each question as honestly as you can. You should think about the amount of difficulty that {the patient} has experienced in the past two weeks. If you are not sure what I am asking, stop me and I will explain it to you.

1. "In your own words, what are these instructions telling you to do?"

2. "How easy or difficult are the instructions to understand?"

   Instructions to interviewer: Follow up with probes
   - *If easy:* "What makes them easy to understand?
   - *If difficult:* "What makes them difficult to understand?

3. "How easy or difficult is it to think about the past 2 weeks when thinking about the cognitive difficulties of the {patient}?"

Part 3B: SCoRS item clarity

Interviewer: "Now we will start with the questionnaire – the questionnaire has 20 questions. As you saw in the instructions, I am going to ask you questions about the level of difficulty {the patient, your son/daughter, your roommate…etc.} may experience with certain tasks. The responses for each question are none, mild, moderate, and severe. He/she may have 'no' difficulty or maybe they have 'severe' difficulty with the task. With other tasks you may feel that they have only 'mild' or 'moderate' difficulty. Do your best to answer each question as honestly as you can. You should think about the amount of difficulty that {the patient} has

experienced in the past two weeks. If you are not sure what I am asking, please stop me and I will explain it to you. Do you have any questions?"

Instruction to the interviewer: Make sure to answer all of the informant's questions before proceeding.

Item 1

"The first question of the ScoRS questionnaire is the following: does the {patient} have difficulty remembering names of people they know or meet? For example, roommate, nurse, doctor, family, and friends?"

4. "In your own words, what does this question ask about?"

Instruction to interviewer: Follow up with probes as needed (e.g., "What is it asking you to do?")

5. Now we would like to talk about response options for this question. The response options are:

None: No difficulties
Mild: Remembers most names of people that he/she knows but not all of the people he/she has just met
Moderate: Forgets many names of people he/she knows and all of the names of people he/she has just met
Severe: Forgets all or almost all names of people he/she knows and meets

''How easy or difficult is it to pick a response?''

"Now we are moving on to the next question."

Item 2

"Question 2 of the SCoRS is the following: does the {patient} you care for have difficulty remembering how to get places? For example, how to get to the restroom, their own room, a friend's house?"

6. "In your own words, what does this question ask about?"

Instruction to interviewer: Follow up with probes as needed (e.g., "What is it asking you to do? How helpful are the provided examples?")

7. Now we would like to talk about response options for this question. The response options are:

None: No difficulties
Mild: Forgets infrequently
Moderate: Is only able to get to frequently visited places
Severe: Unable to get anyplace without assistance because difficulties with memory

''How easy or difficult is it to pick a response?''

"Now we are moving on to the next question."

Item 3

"Question 3 of the SCoRS is the following: does the {patient} you care for have difficulty following a TV show? For example, a favorite show or the news."

8. "In your own words, what does this question ask about?"

Instruction to interviewer: Follow up with probes as needed (e.g., "What is it asking you to do? How helpful are the provided examples?")

9. Now we would like to talk about response options for this question. The response options are:

None: No difficulties
Mild: Can only follow a short movie or news show
Moderate: Can only follow a light, 30 minutes show (i.e. sitcom)
Severe: Unable to follow a TV show for any period of time

''How easy or difficult is it to pick a response?''

"Now we are moving on to the next question."

Item 4

"Question 4 of the SCoRS is the following: does the {patient} you care for have difficulty remembering where they put things? For example, where they put clothes, the newspaper, or cigarettes?"

10. "In your own words, what does this question ask about?"

Instruction to interviewer: Follow up with probes as needed (e.g., "What is it asking you to do? How helpful are the provided examples?")

11. Now we would like to talk about response options for this question. The response options are:

None: No difficulties
Mild: Rare instances of forgetfulness
Moderate: Frequent instances of forgetfulness
Severe: Very frequent instances of forgetfulness or forgetting items of great importance

''How easy or difficult is it to pick a response?''

"Now we are moving on to the next question."

Item 5

"Question 5 of the SCoRS is the following: does the {patient} you care for have difficulty remembering their chores and responsibilities? For example, household chores or appointments?"

12. "In your own words, what does this question ask about?"

Instruction to interviewer: Follow up with probes as needed (e.g., "What is it asking you to do? How helpful are the provided examples?")

13. Now we would like to talk about response options for this question. The response options are:

None: No difficulties
Mild: Infrequently forgets
Moderate: Forgets only those things that do not occur everyday
Severe: Forgets all or almost all of his/her responsibilities

‘’How easy or difficult is it to pick a response?’’

“Now we are moving on to the next question.”

Item 6

“Question 6 of the SCoRS is the following: does the {patient} you care for have difficulty learning how to use new gadgets and equipment? For example, computer, washer, microwave, phone, remote, DVR?”

14. “In your own words, what does this question ask about?”

Instruction to interviewer: Follow up with probes as needed (e.g., “What is it asking you to do? How helpful are the provided examples?”)

15. Now we would like to talk about response options for this question. The response options are:

None: No difficulties
Mild: Takes longer to learn than most, but can usually do it
Moderate: Takes longer and needs to be taught; can not learn some things
Severe: Unable to learn how to use new gadgets and equipment

‘’How easy or difficult is it to pick a response?’’

“Now we are moving on to the next question.”

Item 7

“Question 7 of the SCoRS is the following: does the {patient} you care for have difficulty remembering information and/or instructions recently given to them? For example, telephone numbers, directions, names?”

16. “In your own words, what does this question ask about?”

Instruction to interviewer: Follow up with probes as needed (e.g., "What is it asking you to do? How helpful are the provided examples?")

17. Now we would like to talk about response options for this question. The response options are:

None: No difficulties
Mild: Rarely has difficulty remembering information
Moderate: Frequently forgets information given
Severe: Almost always forgets information

''How easy or difficult is it to pick a response?''

"Now we are moving on to the next question."

Item 8

"Question 8 of the SCoRS is the following: does the {patient} you care for have difficulty remembering what they were going to say? For example, forgetting words, or stopping mid-sentence?"

18. "In your own words, what does this question ask about?"

Instruction to interviewer: Follow up with probes as needed (e.g., "What is it asking you to do? How helpful are the provided examples?")

19. Now we would like to talk about response options for this question. The response options are:

None: No difficulties
Mild: Rare instances of forgetfulness when speaking
Moderate: Frequent instances of forgetfulness when speaking
Severe: Frequency of forgetfulness makes communication very difficult

''How easy or difficult is it to pick a response?''

"Now we are moving on to the next question."

Item 9

"Question 9 of the SCoRS is the following: does the {patient} you care for have difficulty keeping track of their money? For example, managing bills or counting change?"

20. "In your own words, what does this question ask about?"

Instruction to interviewer: Follow up with probes as needed (e.g., "What is it asking you to do? How helpful are the provided examples?")

21. Now we would like to talk about response options for this question. The response options are:

None: No difficulties
Mild: Some difficulty but can usually do it
Moderate: Significant difficulty either with counting change or paying bills
Severe: Unable to keep track of his/her money because of cognitive difficulties

''How easy or difficult is it to pick a response?''

"Now we are moving on to the next question."

Item 10

"Question 10 of the SCoRS is the following: does the {patient} you care for have difficulty keeping their words from being jumbled together? For example, words get mixed up or "run together"?"

22. "In your own words, what does this question ask about?"

Instruction to interviewer: Follow up with probes as needed (e.g., "What is it asking you to do? How helpful are the provided examples?")

23. Now we would like to talk about response options for this question. The response options are:

None: No difficulties
Mild: Sometimes will jumble words but it's rare
Moderate: Can have a conversation but jumbles words frequently

Severe: Unable to have a conversation due to jumbled words

''How easy or difficult is it to pick a response?''

"Now we are moving on to the next question."

Item 11

"Question 11 of the SCoRS is the following: does the {patient} you care for have difficulty concentrating well enough to read a newspaper or a book? For example, reading the same sentence or the same page over and over?"

24. "In your own words, what does this question ask about?"

Instruction to interviewer: Follow up with probes as needed (e.g., "What is it asking you to do? How helpful are the provided examples?")

25. Now we would like to talk about response options for this question. The response options are:

None: No difficulties
Mild: Can concentrate except for rare occasions
Moderate: Can concentrate on short and easy to understand materials
Severe: Unable to read even the simplest materials due to concentration problems

''How easy or difficult is it to pick a response?''

"Now we are moving on to the next question."

Item 12

"Question 12 of the SCoRS is the following: does the {patient} you care for have difficulty with familiar tasks? For example cooking, driving, showering, getting dressed?"

26. "In your own words, what does this question ask about?"

Instruction to interviewer: Follow up with probes as needed (e.g., "What is it asking you to do? How helpful are the provided examples?")

27. Now we would like to talk about response options for this question. The response options are:

None: No difficulties
Mild: Rarely has difficulty completing the task
Moderate: Frequently needs verbal assistance to complete the task
Severe: Needs physical assistance to do these tasks due to cognitive difficulties

''How easy or difficult is it to pick a response?''

"Now we are moving on to the next question."

Item 13

"Question 13 of the SCoRS is the following: does the {patient} you care for have difficulty staying focused? For example, daydreaming, trouble paying attention to someone talking?"

28. "In your own words, what does this question ask about?"

Instruction to interviewer: Follow up with probes as needed (e.g., "What is it asking you to do? How helpful are the provided examples?")

29. Now we would like to talk about response options for this question. The response options are:

None: No difficulties
Mild: Sometimes unable to stay focused
Moderate: Frequently unable to stay focused
Severe: Almost always unable to stay focused

''How easy or difficult is it to pick a response?''

"Now we are moving on to the next question."

Item 14

"Question 14 of the SCoRS is the following: does the {patient} you care for have difficulty learning new things? For example, new words, new ways of doing things, new schedules?"

30. "In your own words, what does this question ask about?"

Instruction to interviewer: Follow up with probes as needed (e.g., "What is it asking you to do? How helpful are the provided examples?")

31. Now we would like to talk about response options for this question. The response options are:

None: No difficulties
Mild: Takes longer to learn than most, but can usually do it
Moderate: Takes longer and needs special attention
Severe: Unable to learn almost all new things

''How easy or difficult is it to pick a response?''

"Now we are moving on to the next question."

Item 15

"Question 15 of the SCoRS is the following: does the {patient} you care for have difficulty speaking as fast as they would like? For example, slow speech or pauses?"

32. "In your own words, what does this question ask about?"

Instruction to interviewer: Follow up with probes as needed (e.g., "What is it asking you to do? How helpful are the provided examples?")

33. Now we would like to talk about response options for this question. The response options are:

None: No difficulties
Mild: Rarely speaks slowly because of cognitive difficulties
Moderate: Often speaks slowly because of cognitive difficulties

Severe: Ability to converse is jeopardized because of cognitive difficulties

‘‘How easy or difficult is it to pick a response?’’

“Now we are moving on to the next question.”

Item 16

“Question 16 of the SCoRS is the following: does the {patient} you care for have difficulty doing things quickly? For example, writing or lighting a cigarette?”

34. “In your own words, what does this question ask about?”

Instruction to interviewer: Follow up with probes as needed (e.g., “What is it asking you to do? How helpful are the provided examples?”)

35. Now we would like to talk about response options for this question. The response options are:

None: No difficulties
Mild: Slightly slower than normal pace
Moderate: Significantly slower; may need prompting to do things quickly
Severe: Unable to get things done because time runs out

‘‘How easy or difficult is it to pick a response?’’

“Now we are moving on to the next question.”

Item 17

“Question 17 of the SCoRS is the following: does the {patient} you care for have difficulty handling changes in their daily routine? For example, appointments, special visits, or group therapy?”

36. “In your own words, what does this question ask about?”

Instruction to interviewer: Follow up with probes as needed (e.g., “What is it asking you to do? How helpful are the provided examples?”)

37. Now we would like to talk about response options for this question. The response options are:

None: No difficulties
Mild: Can adjust with considerable effort
Moderate: Will eventually adjust with assistance
Severe: Changes in the daily routine are impossible

''How easy or difficult is it to pick a response?''

"Now we are moving on to the next question."

Item 18

"Question 18 of the SCoRS is the following: does the {patient} you care for have difficulty understanding what people mean when they are talking to them? For example, feeling confused by what someone says?"

38. "In your own words, what does this question ask about?"

Instruction to interviewer: Follow up with probes as needed (e.g., "What is it asking you to do? How helpful are the provided examples?")

39. Now we would like to talk about response options for this question. The response options are:

None: No difficulties
Mild: Some difficulty understanding what people mean
Moderate: Often has difficulty understanding what people mean
Severe: Frequently unable to understand what people mean

''How easy or difficult is it to pick a response?''

"Now we are moving on to the next question."

Item 19

“Question 19 of the SCoRS is the following: does the {patient} you care for have difficulty understanding how other people feel about things? For example, misunderstanding people’s emotions by their facial expressions or tone of their voice?”

40. “In your own words, what does this question ask about?”

Instruction to interviewer: Follow up with probes as needed (e.g., “What is it asking you to do? How helpful are the provided examples?”)

41. Now we would like to talk about response options for this question. The response options are:

None: No difficulties
Mild: Rarely has difficulty understanding how people feel
Moderate: Often has difficulty understanding how people feel
Severe: Very frequent instances of difficulty understanding how people feel

‘‘How easy or difficult is it to pick a response?’’

“Now we are moving on to the next question.”

Item 20

“Question 20 of the SCoRS is the following: does the {patient} you care for have difficulty following conversations in a group? For example, participation, able to follow a conversation?”

42. “In your own words, what does this question ask about?”

Instruction to interviewer: Follow up with probes as needed (e.g., “What is it asking you to do? How helpful are the provided examples?”)

43. Now we would like to talk about response options for this question. The response options are:

None: No difficulties
Mild: Few difficulties following conversations in a group
Moderate: Often unable to follow conversations in a group
Severe: Frequently unable to follow conversations in a group and communication in that setting is difficult or impossible

''How easy or difficult is it to pick a response?''

"Now we are moving on to some more instructions."

44. We would also like to know the overall degree of severity of cognitive impairment of the person with schizophrenia that you care for and had in mind during this interview (considering all aspects discussed).

Please choose the response below that best describes the degree of severity of the person's cognitive impairment in the past two weeks.

☐ *None*

☐ *Mild*

☐ *Moderate*

☐ *Severe*

45. Finally, we would like to know whether you think there are any important symptoms or impacts of cognitive impairments that should be added or removed from the SCoRS questionnaire?

Instruction to the interviewer: If the respondent says 'Yes' to adding to removing items from SCoRS, and the respondent to explain why they think items should be added or removed.

Part 3C: SCoRS follow up instruction clarity & informants rating

Instruction to interviewer: Read the bolded text in the instruction box below. Prompt/probe as necessary to help establish and clarify proper timeframe.

Interviewer: "Now I'm going to read the next instructions in the SCoRS questionnaire to you. This questionnaire will be used in clinical trials to interview people like you, who care for or work with schizophrenia patients and know them well, in order to assess how much the patient's cognitive functioning has improved during the clinical trial. Imagine, your patient/the person you are caring for, has just completed his/her participation in a clinical trial and had tested a new mediation. Can you please listen to the instructions I am going to read now? Once I'm done reading the instructions, I will ask you a few questions."

Informant Instructions:
Please read the following **bolded** text, verbatim. Circle answer.
I want you to think about how difficult it was for {the patient, your son/daughter, your roommate…etc.*} to perform these tasks since they started taking the study medication. [Prompt/probe as necessary to help establish and clarify proper timeframe.]. Now I want you to think about how difficult it is for him/her to do the things we just discussed today. Rate how their level of difficulty with these tasks has changed. I want you to do this using a scale of 1-7 with 1 being "much worse", 4 being "the same", and 7 being "much better".

46. *"*In your own words, what are these instructions telling people to do?"

47. *"*How easy or difficult are the instructions to understand?"

Instructions to interviewer: Follow up with probes

- *If easy:* "What makes them easy to understand?
- *If difficult:* "What makes them difficult to understand?

Interviewer: "Now we will look at the scale."

"Please look at the scale and I will ask you a few questions about it."

much worse	INFORMANT RATES					much improved
1	2	3	4	5	6	7
	moderately. worse	minimally worse	no change	minimally. improved	moderately improved	

48. "In your own words, what does "much improved" mean to you?"

49. "In your own words, what does "moderately improved" mean to you?"

50. "In your own words, what does "minimally improved" mean to you?"

51. "In your own words, what does "no change" mean to you?"

52. "In your own words, what does "minimally worse" mean to you?"

53. "In your own words, what does "moderately worse" mean to you?"

54. "In your own words, what does "much worse" mean to you?

55. "How easy or difficult is it to pick an answer along the scale?"

Instructions to interviewer: Follow up with probes
- *If easy:* "What makes it easy?
- *If difficult:* "What makes it difficult?

"This was the final question. Thank you very much for participating in this study. We very much appreciate your answers. Do you have any final comments that you would like to share before we end the interview?"
